# Effects of Foot-Core Training on Foot-Ankle Kinematics and Running Kinetics in Runners: Secondary Outcomes From a Randomized Controlled Trial

**DOI:** 10.3389/fbioe.2022.890428

**Published:** 2022-04-14

**Authors:** Alessandra B. Matias, Ricky Watari, Ulisses T. Taddei, Paolo Caravaggi, Rafael S. Inoue, Raissa B. Thibes, Eneida Y. Suda, Marcus F. Vieira, Isabel C. N. Sacco

**Affiliations:** ^1^ Faculdade de Medicina, Physical Therapy, Speech and Occupational Therapy Department, Universidade de São Paulo, Sao Paulo, Brazil; ^2^ Movement Analysis Laboratory, IRCCS Istituto Ortopedico Rizzoli, Bologna, Italy; ^3^ Center of Mathematics, Computing and Cognition, Universidade Federal do ABC, Santo André, Brazil; ^4^ Bioengineering and Biomechanics Laboratory, Federal University of Goiás, Goiás, Brazil

**Keywords:** running, exercise therapy, rehabilitation research, foot joint kinematics, running injuries, statistical parametric mapping

## Abstract

This study investigated the effectiveness of an 8-week foot-core exercise training program on foot-ankle kinematics during running and also on running kinetics (impact loads), with particular interest in biomechanical outcomes considered risk factors for running-related injuries in recreational runners. A single-blind, randomized, controlled trial was conducted with 87 recreational runners randomly allocated to either the control (CG) or intervention (IG) group and assessed at baseline and after 8 weeks. The IG underwent foot-core training 3 times/week, while the CG followed a placebo lower-limb stretching protocol. The participants ran on a force-instrumented treadmill at a self-selected speed while foot-segment motion was captured simultaneously with kinetic measurements. After the intervention, there were statistically significant changed in foot biomechanics, such as: IG participants strike the ground with a more inverted calcaneus and a less dorsiflexed midfoot than those in the CG; at midstance, ran with a less plantarflexed and more adducted forefoot and a more abducted hallux; and at push-off, ran with a less dorsiflexed midfoot and a less adducted and more dorsiflexed hallux. The IG runners also had significantly decreased medial longitudinal arch excursion (*p* = 0.024) and increased rearfoot inversion (*p* = 0.037). The 8-week foot-core exercise program had no effect on impact (*p* = 0.129) and breaking forces (*p* = 0.934) or on vertical loading rate (*p* = 0.537), but it was positively effective in changing foot-ankle kinematic patterns.”

## Introduction

Running is one of the most popular sports and fitness activities worldwide owing to its simple requirements in terms of gear and ability to be performed indoors or outdoors. However, one of the drawbacks is the high incidence of running-related injuries (RRI), such as patellofemoral pain syndrome, iliotibial band friction syndrome, plantar fasciitis, meniscal injuries and patellar tendinopathy (Van Gent et al., 2007; Kluitenberg et al., 2015). The etiology of RRI is believed to be multifactorial (Buist et al., 2010; van der Worp et al., 2015; Davis et al., 2017; Dudley et al., 2017) and is generally thought to include the following biomechanical risk factors: altered medial longitudinal arch (MLA) posture (Cowan et al., 1993; Busseuil et al., 1998; Bennett et al., 2001; Williams et al., 2001; Weist et al., 2004; Headlee et al., 2008; Kelly et al., 2015); greater ankle (Messier and Pittala, 1988; Willems et al., 2006; Pohl and Buckley, 2008) or rearfoot (Noehren et al., 2007, 2013; Hein and Grau, 2014; Messier et al., 2018) eversion; and higher loading rates (Hreljac et al., 2000; Milner et al., 2005; Zadpoor and Nikooyan, 2011; Bredeweg et al., 2013; Davis et al., 2016), impact peaks (Bennell et al., 1996; Hreljac et al., 2000; Milner et al., 2005; Davis et al., 2016), and breaking forces (Grimston et al., 1991; Messier et al., 1995; Napier et al., 2018).

Several therapeutic strategies have been implemented in recent decades to minimize RRI incidence, but these have yielded poor outcomes (Buist et al., 2008; Fokkema et al., 2017; Hespanhol et al., 2018). Some of the most commonly adopted therapeutic approaches to reducing RRI are strengthening programs focused on the hip and the core areas—i.e., the “top-down” approach (Powers, 2010; Hott et al., 2015; Palmer et al., 2015). This approach claims that increased hip and core muscle (abdominal and multifidus muscles) strength contribute to the reduction of non-sagittal joint movements and moments, and thus of the loads in the adjacent joints in the lower limbs, which in turn would result in lower risks of RRI (Fredericson and Moore, 2005; Brumitt, 2009; Snyder et al., 2009; Powers, 2010; Hott et al., 2015; Palmer et al., 2015). Although this approach is very popular (Johnston et al., 2003; Fredericson and Moore, 2005; Brumitt, 2009; Willy and Davis, 2011), its beneficial effects in diminishing the incidence and the biomechanical risk factors of RRI (Ceyssens et al., 2019) are yet to be proven (Nigg et al., 2017).

A promising alternative strategy, the so-called “bottom-up” approach, targeted foot core muscles strength (intrinsic and extrinsic foot muscles) (McKeon and Fourchet, 2015a) and biomechanics with the goal of attenuating mechanical loads directly related to RRI (Milner et al., 2005; Warden et al., 2008; Davis et al., 2016). It applies the lumbopelvic core system concept to the foot core system. The lumbopelvic core system is comprised of interacting subsystems (neural, passive and active) that provide relevant sensory input and functional stability for accommodating to changing demands during both static and dynamic activities (McKeon et al., 2015b). The application of this concept to the foot core it is logical as it works just like the trunk core considering that the subsystems in the foot also provide a stable base on which the primary movers of the foot-ankle complex, those with larger cross-sectional areas and moment arms, can act to cause gross motion, and the intrinsic muscles work as the local stabilizers, as they have small cross-sectional areas and small moment arms (McKeon and Fourchet, 2015a; McKeon et al., 2015b). According to the “bottom-up” theoretical assumptions (Tiberio, 1987; Feltner et al., 1994; Hollman et al., 2006; Lucas-Cuevas et al., 2016; Nigg et al., 2017), this approach may potentially change the mechanical or biomechanical response of more proximal joints (knee, hip). The foot is a biomechanically complex structure made of 26 bones, four layers of plantar intrinsic muscles, and several joints, providing the foot with multiple degrees of freedom. Active and passive elements in the foot, such as ligaments and soft tissues, act in synergy to make the foot a mobile adapter capable of receiving and attenuating external loads, and of storing and releasing elastic energy (Kelly et al., 2018; Farris et al., 2019). Hypothetically, a stronger foot structure (stronger foot muscles and improved mechanical properties of passive tissues—tendons, ligaments and joint tissues) and the medial longitudinal arch should better dissipate excessive and cumulative loads through actively supporting changing the function of the foot from a dampener in the early stance to a spring in the late stance (Ker et al., 1987; Taddei et al., 2020a). Some studies demonstrate the benefits of strengthening the foot core muscles and, knowing the intrinsic foot muscle’s role in dampening impacts and propelling the body during running (Ker et al., 1987; Kluitenberg et al., 2015; Taddei et al., 2020b), it is logical to think that these roles were also improved with this “bottom-up” training (Feltner et al., 1994; Nigg et al., 1997, 2017; Matias et al., 2016; Baltich et al., 2017; Mølgaard et al., 2018). Thus, we can assume that by reducing shock, cumulative load, better controlling foot-ankle motion and alignment, strengthening the foot muscles resulted in preventing the RRI in the intervention group.

There is some evidence that this approach is effective in preventing RRI and promoting functional gains related to running. A previous proof-of-concept study performed by our group showed that an 8-week foot-core strengthening program increased the intrinsic anatomical cross-sectional area of the foot muscle and the propulsive impulse during running (Taddei et al., 2018) The primary outcome of our single-blind, randomized, controlled trial (RCT) concerning RRI prevention showed that 8 weeks of foot-core training in healthy runners resulted in a 2.42-fold reduction of RRI incidence at the 1-year follow-up compared with a placebo stretching program (Taddei et al., 2020b). There was also a significant correlation between time-to-injury and foot strength gain that all might support the hypothesis-driven mechanism we described, where the stronger the runner’s foot, the longer it took the runner to develop an RRI. In this follow-up report of secondary outcomes from that study, we report the effects of the 8-week foot-core exercise training program (Taddei et al., 2020b) on the participants who had their foot-ankle kinematics and running kinetics assessed, with particular interest in the biomechanical outcomes considered to be risk factors for RRI in recreational runners.

## Materials and Methods

A detailed protocol of the single-blind RCT with two parallel arms has been published elsewhere (Matias et al., 2016). The study was approved by the Ethics Committee of the School of Medicine of the University of São Paulo (18/03/2015, Protocol #031/15), and was registered with clinicaltrials.gov (Identifier NCT02306148).

### Participants and Recruitment

Adult recreational runners were recruited through digital social media advertising, posted flyers, and direct contact with runners and running groups in the university surroundings between August 2015 and August 2017. All participants were RRI-free in the 2 months prior to the baseline assessment, had no experience running barefoot or in minimalist shoes, were without chronic diseases or impairments that could influence running performance, and had run between 20 and 100 km/week for ≥1 year.

### Sample Size

In our previous study (Taddei et al., 2018), an a priori sample size was calculated using several kinematic foot outcomes. The fifth metatarsal bone to the ground (V2G), second metatarsal bone to the ground (S2G), first metatarsal bone to the ground (F2G), second to first metatarsal bone divergence in the transverse plane of the foot (S2F), second to fifth metatarsal bone divergence in the transverse plane of the foot (S2V) and the medial longitudinal arch (MLA) required 38, 86, 58, 2,184, 34, and 6 participants, respectively. Based on 80% power and a significance level of 5%, the study indicated that we needed a total of 86 participants for most of the secondary outcomes, and we included 87 participants in the present study as they had their running biomechanics assessed. It was a sample size feasible to obtain from the full sample of the RCT (*n* = 119) and it would be capable of detecting changes in almost all foot biomechanical outcomes, except the one that needed more than two thousand participants, what would make the study unfeasible.

### Randomization and Follow-Up Assessments

After the runners’ agreement to participate and completion of the baseline questionnaire, they were randomized into either the IG or the CG by using Clinstat software (University of York, Heslington, UK) to generate a randomization list with blocks of eight. The randomization list was developed by an individual who is not part of the research team. The codes for the groups were kept in opaque, sealed envelopes numbered from 1 to 120, and the researchers involved in the allocation and assessments were blind to the group codes and block size. The participants were enrolled and assigned to the interventions by a member of the research group. From the 119 participants included in the full RCT to evaluate RRI incidence over 1-year follow up, the 87 participants that had their running biomechanics assessed were included in the current analysis of secondary outcomes, 41 in the IG and 46 in the CG.

The trial statistician was blind to treatment allocation until the main analysis had been completed. All participants’ data were kept confidential before, during, and after the study by encoding their names.

Participants allocated to the IG were given access to 8 weeks of a training program. Participants in the CG were informed about their allocation into the control group and were instructed to perform a 5-minute static stretching protocol (Matias et al., 2016) as a placebo. We instructed the participants to keep their allocation group information strictly personal.

The baseline questionnaire consisted of six sections (demographics, training, running events, Foot Health Status Questionnaire, anthropometrics, and previous RRIs) (Table 1). The follow-up questionnaires asked about running routine, adherence to the foot-core training program, and RRIs.

**TABLE 1:** Baseline questionnaire and follow-up.

Questionnaire	Section	Items
Baseline questionnaire	Demographics	Sex
		Age
		Body mass (kg)
		Height (m)
		Body Mass Index (kg/m^2^)
	Training	Running experience (years)
		Average running frequency over the last month (times per week)
		Average running distance over the last month (km/week)
		Average pace over the last month (min/km)
	Running events	Member of athletic association (yes)
		Previous participation in running events (yes/no)
		Average participations in running events before
	Foot health status Questionnaire	Eight domains of the questionnaire
	Anthropometrics	Foot posture index
		Cavanagh Rodgers index
	Previous running-related injuries	Running-related injury in previous 12 months (yes/no)
		Location of running injury
Weekly follow-up questionnaires	Training	Running frequency (times/week)
		Running distance (km/week)
	Intervention protocol sessions	Number of foot exercise sessions completed
	New running-related injuries	New running-related injury since filling in previous questionnaire (yes/no)
		Location of new running-related injury
		Time to injury

### Intervention

The foot-core training program to prevent RRI focused on the foot and ankle muscles, with 12 exercises progressing weekly in volume and difficulty (Matias et al., 2016). Participants in the IG were trained once a week by a physiotherapist and given online access to web-based software developed for this project with descriptions of the exercises and videos to help them perform the same exercises an additional 3×/week, remotely supervised by the same physiotherapist. Each session, either locally or remotely supervised, had a duration of 20–30 min. Gradual and progressive difficulty were offered to the runner, respecting any limitation due to pain, fatigue and/or decrease in performance during execution. The runners in the IG were asked to access the web software daily, entering their data regarding performance of the foot exercise training and ranking their level of difficulty in each exercise from 0 to 10. If the effort score ranged from 0 to 5 and the runner’s performance of each exercise was found adequate during the supervised session by the physiotherapist, the exercises increased in difficulty. If the effort score ranged from 6 to 7, the exercise did not increase in difficulty and no progressions were done on that exercise. Thus, the runner remained in the same exercise progression until he/she scored 0 to 5 in that exercise. Finally, if an IG runner reports a score from 8 to 10, the exercise decreased in difficulty, if possible, until the runner was able to perform it without pain or discomfort.

Runners allocated to the CG received a 5-minute placebo warm-up and muscle stretching exercise routine that should be performed immediately before each running practice. The placebo exercises were developed based on the runner’s habitual routine warm-up combined with muscle stretching exercises focused on the lower limb’s muscles (triceps surae, quadriceps, hamstrings, gluteus) involving both open and closed- kinetic chain exercise. The CG exercises aimed to not have any effect on foot muscles strength and functionality, lower extremity biomechanics or injury prevention. CG runners received weekly feedback and interaction with the physiotherapist through the web-software and calls.

Both groups were instructed to perform their respective exercises 3×/week up to the end of the 1-year follow-up and, to improve adherence to the programs, to register their adherence in the web software. The importance of adhering to the program was reinforced at every contact with the participants. The participants were strongly advised not to engage in any new exercise program during the intervention period.

### Measurements

Eighty-seven participants that had their running biomechanics assessed were included in the current analysis of secondary outcomes. Biomechanical data were collected using an eight-camera motion capture system (Vicon Motion System Ltd., Oxford Metrics, UK) for the acquisition of 3D kinematic data at 200 Hz while running. Sixteen reflective skin markers (each 9 mm in diameter) were placed on the shank and foot in accordance with the Rizzoli multi-segment foot model (Leardini et al., 2007; Portinaro et al., 2014). Following a standing calibration trial, the participants were requested to run barefoot at a self-selected comfortable speed on an AMTI™ force-sensing tandem treadmill (AMTI, Watertown, MA, United States) for the acquisition of ground reaction force data at 1,000 Hz. In order to habituate to the treadmill and to warm up, the participants were instructed to run for 2–3 min before the data collection. A 30-s running trial was recorded at the self-selected comfortable speed after the accommodation period. Heel strike and toe off were identified when the vertical ground reaction force crossed a 30 N threshold. Kinematic and ground reaction force data were filtered using a fourth-order, zero-lag, low-pass Butterworth filter with cut-off frequencies of 10 and 80 Hz, respectively. The outputs of the Rizzoli foot model were calculated by custom-made scripts in Visual3D (Visual3D, C-Motion, Germantown, MD, United States) in accordance with the published definitions (Leardini et al., 2007; Portinaro et al., 2014; Caravaggi et al., 2019). Joint rotations were calculated by using the Joint Coordinate System (Grood and Suntay, 1983) convention. The axes of each joint reference frame were defined as follows: sagittal-plane rotations around the z-axis (medio-lateral); frontal-plane rotations around the x-axis (anterior-posterior); and transverse-plane rotations around the y-axis (vertical**).** Data were normalized to 0–100% of stance phase**.**

### Outcomes

This study is an analysis of the secondary outcomes from the developed RCT. The primary outcome variable was incidence of RRI in recreational runners over the course of a 1-year follow-up and was published elsewhere (Taddei et al., 2020b). The secondary outcomes were related to foot-ankle kinematics during running and running kinetics which were evaluated 8 weeks after the baseline assessment. The foot time series kinematic variables were 3D MLA (Caravaggi et al., 2019) excursion and rotation angles in the three anatomical planes (Sha-Cal, Cal-Mid, Mid-Met, Cal-Met, and Met-Hal). The following metatarsal bone angles were also assessed: sagittal-plane inclination of F2G, S2G, and V2G and transverse-plane divergence between S2F and between S2V. In addition, kinematic and kinetic biomechanical-related risk factors for RRI were investigated as discrete parameters: rearfoot angle (Sha-Cal frontal angle peaks), MLA ROM (max-min), vertical average loading rate (average slope of the line through the interval between 20 and 80% of the time from the foot contact and the first peak), horizontal breaking forces (maximum posterior force, horizontal component), and vertical impact peak (local maximum vertical force at initial contact).

### Statistical Analysis

All analyses used the full set of randomly assigned participants under the intention-to-treat assumption. The generalized linear mixed model (GLMM) method was used for univariate analyses, considering the following as factors: groups (CG and IG); time of assessment (baseline and after 8 weeks); and the interaction effect (time by group), which was our primary outcome comparison. Participants and time were considered as random effects and groups as fixed effects in the GLMM modeling. Q-Q graphs were plotted to verify the adequacy (normality) of each model. Univariate comparisons (main and interaction effects) of the estimated marginal means were adjusted with the Bonferroni correction. The comparisons between the pairs of estimated marginal means were made based on the original scale of each of the dependent variables of the study. Statistical analyses were performed using the Statistical Package for the Social Sciences (SPSS, IBM; v.26.0), adopting a 5% significance level. Cohen’s d effect size was calculated for discrete variables, and effects between 0.2 and 0.5 were considered small, between 0.5 and 0.8 were medium, and above 0.8 were large (Cohen, 1988).

Additionally, to capture features of the entire time series, a vector field analysis of the resultant angles was conducted using one-dimensional statistical parametric mapping (1D-SPM), as described elsewhere (Pataky et al., 2013, 2017). Custom-written MATLAB code (MATLAB 2020a; MathWorks, Natick, United States), using the source code available at http://www.spm1d.org/, was employed in the analysis. The 1D-SPM captures features of the entire time series, rather than a few discrete variables, and can provide additional information. Each component of each time series was interpolated to contain 101 points (0–100% of the stance phase) and organized in an array with two or three corresponding matrices, one for each variable component; 87 rows, one for each subject; and 101 columns. 1D-SPM ANOVA followed by post-hoc SPM t-tests was used for 1D variables (F2G, S2G, V2G, S2F, S2V, and MLA). Paired (for assessment comparisons) and independent (for group comparisons) Hotelling’s T2 tests were used for comparison of 3D variables (Sha-Cal, Cal-Mid, Mid-Met, Cal-Met, and Met-Hal) in a 3D vector field SPM analysis, followed by the paired or independent t-test as a post-hoc test with a Sidák correction. The output of SPM provides T2, F, and t values for each sample of the investigated kinematic time series, and the threshold corresponding to the set alpha level (see Supplementary Figures). The T2, F, and t values exceeding this threshold (marked as black bars below each figure, e.g., in Figures 2, 3) indicate significant differences in the corresponding portion of the time series (Figures 2–5).

## Results and Discussion

**FIGURE 1:**
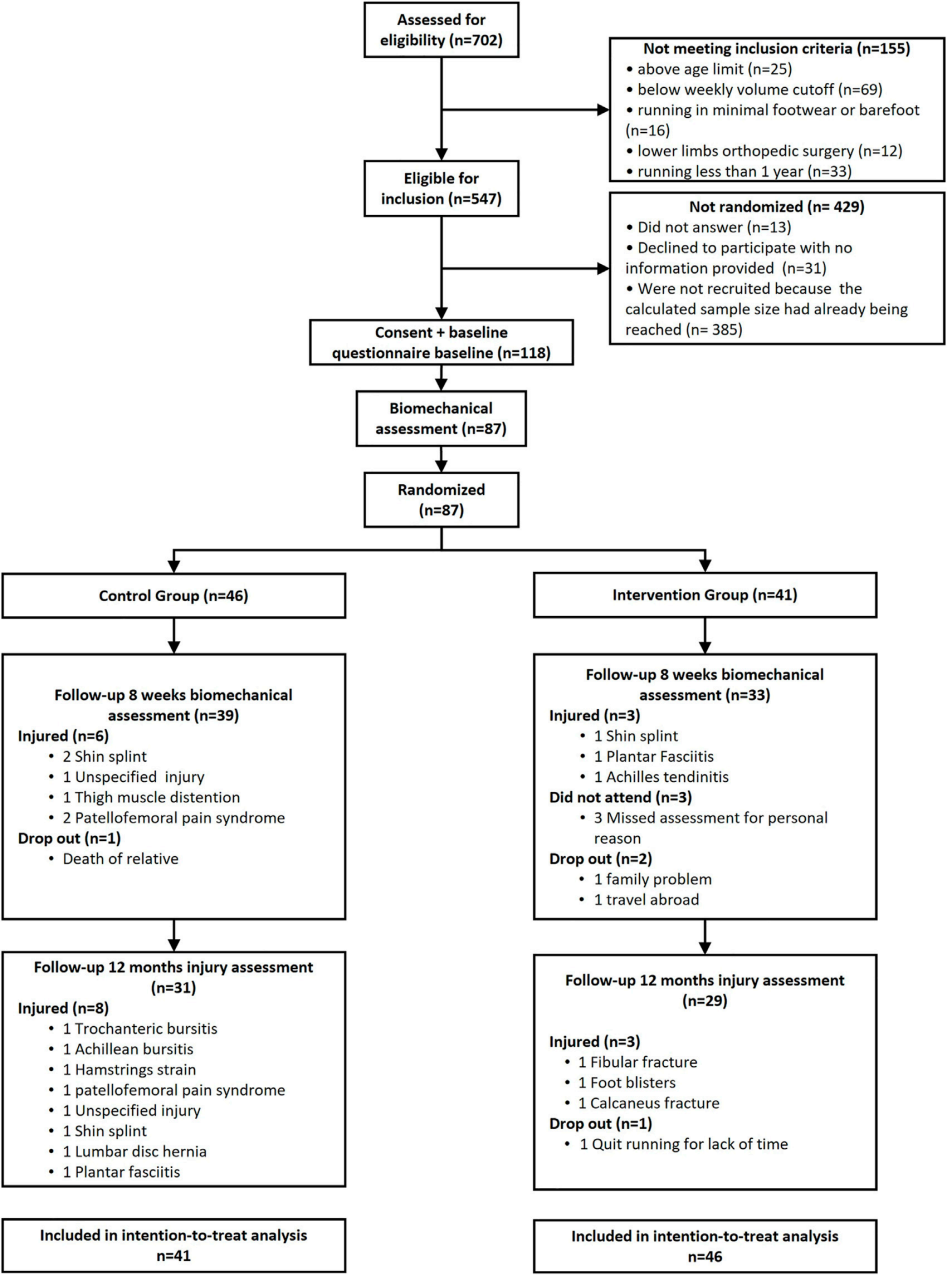
Flowchart of recruitment, assessment, and follow-up process.

Baseline assessment data are described in Table 2. The participants were randomly assigned to either the control group (CG) or to the 8-week supervised foot-core training group (interventional group, IG) (Figure 1). They were on average 40.3 (SD 6.9) years old, and the majority (51.2%) were female (Table 2), with a mean running experience of 6.5 (SD 5.7) years, a median Foot Posture Index of 2.0 (8% highly supinated, 26% supinated, 49% normal, 14% pronated, 1% highly pronated). An RRI was reported by 46% of all runners in the 12 months prior to their participation in the study (Table 2). An RRI was reported by 46% of all runners in the 12 months prior to their participation in the study (Table 2). The mean running volume at baseline was 35.8 (SD 27.6) km/week (Table 2). During the follow-up assessments and contact with the runners throughout the study, we certified that they followed our instructions rigorously about any changes in their regular physical activity, such as use of minimalist shoes, barefoot sports, or isolated foot strengthening. All participants reported the modifications on their sports activities, and it was not observed any activities that would modify the biomechanical outcomes. Participants were recommended to maintain their running routine during the study period, which was closely monitored to ensure that participants and groups did not differ significantly in the volume run each week during the 8-week intervention period (all participants: mean volume of 83.72 (SD 59.66) km/week; IG: 77.78 (SD 57.31) km/week, CG: 89.09 (SD 61.90) km/week, *p* = 0.40).

**TABLE 2:** Baseline characteristics of participants from the intervention and control groups.

	All participants		Intervention group		Control group	
	N	%/Mean (SD)	N	%/Mean (SD)	N	%/Mean (SD)
N	87		41	47.1%	46	52.9%
Demographics						
Sex (male)	42	48.8%	17	41.5%	25	54.3%
Age (years)		40.3 (6.9)		40.3 (7.7)		40.3 (6.1)
Body mass (kg)		70.5 (13.1)		67.2 (12.1)		73.5 (13.0)
Height (m)		169.3 (8.8)		166.5 (7.6)		171.8 (9.0)
Body Mass Index (kg/m^2^)		24.5 (3.2)		24.1 (3.0)		24.8 (3.3)
Training						
Running Experience (years)		6.5 (5.7)		5.9 (5.1)		7.1 (6.2)
Running frequency per week		3.7 (1.0)		3.8 (1.0)		3.6 (1.2)
Running volume per week (km)		35.8 (27.6)		31.7 (22.5)		39.4 (30.8)
Average pace (min/km)		6.58” (1.36)		6.46” (2.36)		6.69” (2.38)
Running event						
Member of athletic association (yes)	38	43.7%	19	46.3%	19	41.3%
Participated in a running event before (yes)	83	95.4%	40	97.6%	43	93.5%
Number of running events before		37.0 (41.7)		29.3 (31.8)		44.0 (47.5)
Anthropometrics						
Foot posture index—median (25th and 75th percentiles)		2.0 (−2.25; 4.0)		2.0 (−3.0; 4.0)		1.0 (−1.0; 4.0)
Cavanagh & Rodgers index (right foot)		0.20 (0.06)		0.22 (0.05)		0.18 (0.07)
Previous RRI						
Previous RRI in previous 12 months (yes)	40	46.0%	20	48.8%	20	43.5%
FHSQ score (0-100 points)						
Foot pain		90.5 (12.7)		89.9 (13.3)		91.6 (12.0)
Foot function		98.2 (6.0)		98.8 (5.0)		97.6 (6.6)
Shoes		74.5 (24.8)		73.4 (26.9)		76.8 (22.3)
General Foot Health		78.4 (22.9)		76.4 (25.0)		80.3 (20.4)
General Health		86.2 (13.4)		87.1 (12.9)		85.1 (13.6)
Physical Activity		95.5 (15.3)		95.1 (15.0)		95.8 (15.3)
Social Activity		87.5 (15.0)		88.4 (14.2)		86.7 (15.5)
Vigor		75.2 (13.5)		74.1 (11.8)		76.1 (14.4)
Running Biomechanics						
Medial Longitudinal Arch ROM (deg)		3.40 (7.39)		6.16 (8.14)		3.59 (7.89)
Sha-Cal Inv (-) Peak (deg)		–3.12 (7.38)		–0.56 (7.42)		–3.30 (8.71)
Sha-Cal Eve (+) Peak (deg)		6.81 (2.82)		6.72 (3.29)		6.89 (2.35)
Vertical Impact Peak (BW)		1.14 (0.49)		1.13 (0.39)		1.21 (0.44)
Vertical Average Load Rate (BW*s^−1^)		75.05 (55.75)		75.19 (46.43)		73.48 (43.17)
Peak Braking Force (BW)		–0.24 (0.06)		–0.24 (0.05)		–0.24 (0.05)

FHSQ, foot health status questionnaire; ROM, range of motion; BW, bodyweight; Eve, eversion; Inv, inversion; RRI, running-related injury; Sha-Cal, calcaneus with respect to the shank joint angles.

During the 8-week training program, all participants completed a custom online survey regarding new RRIs (if any had occurred) and completed the remote training sessions. The dropout rate was 4.9% (2 participants) in the IG and 2.2% (1 participant) in the CG. Participants in the IG were expected to attend the locally supervised training with the designated researcher once a week. The full protocol lasted 8 weeks, and participants were excluded if they missed two consecutive weekly sessions. The total adherence to the protocol, defined as attendance at the locally supervised training, was 96.7%, where 100% corresponds to all participants attending all sessions (*n* = 304, after excluding three participants from the IG who suffered injuries during the 8 weeks of training). Adherence to the remote intervention sessions performed by the IG was on average 83.5% between 8 and 16 weeks, 68.5% between 16 and 24 weeks, 62.5% between 24 and 32 weeks, and 48.9% between 32 and 40 weeks (12 months). Both groups, IG and CG, evolved quickly in the exercises and achieved the most difficult level of the proposed exercises during the first 8 weeks of sessions.

Of the 87 runners, 20 had sustained an RRI by the 1-year follow-up: 6 of 41 in the IG, and 14 of 46 in the CG (Figure 1). Injuries in the IG at 1-year follow-up were shin splint, plantar fasciitis, and calcaneal tendinitis. Injuries in the CG were patellofemoral pain, shin splint, and thigh strain.

### The Effect of the Foot-Core Training on Discrete Biomechanical Risk Factors for Running-Related Injuries

The average loading rate, impact, and breaking force peaks were not significantly different between IG and CG at the 8-week follow-up (Table 3). These parameters were chosen as secondary outcomes in this study because they have historically been retrospectively and prospectively associated with RRI. Retrospective studies have shown a strong association between higher vertical loading rates and tibial shock with stress fractures in female runners (Milner et al., 2005), greater vertical impact forces and loading rates with overuse RRI (Hreljac et al., 1999), and higher breaking forces in female runners who sustained an injury in a 15-week period (Napier et al., 2018). Furthermore, in prospective studies, higher impacts and loading rates were observed in runners who sustained an RRI in a 2-year period (Davis et al., 2016) and in novice male runners who sustained an injury in a 9-week period (Bredeweg et al., 2013). However, another prospective study did not find differences in loading rates between injured and uninjured collegiate runners at 12 weeks (Kuhman et al., 2016). The etiology of RRI is multifactorial, and the different types of RRI observed in our RCT after 1 year (14 in the CG and six in the IG) were probably generated by multiple RRI mechanisms. However, establishing a direct relationship between this kinetic risk factor and RRIs would be difficult due to the small sample and the accompanying low statistical power.

**TABLE 3:** Mean (standard deviation) pre- and post-intervention values for kinetic and kinematic biomechanical measures in the experimental groups. *p*-values of the interaction effect (group × time) and Cohen’s d effect sizes are presented.

Variable	Intervention group		Control group		Interaction	Cohen’s d effect size (95% CI)
	*Pre*	*Post*	*Pre*	*Post*	*p*	
MLA ROM (deg)	6.16 (8.14)	0.17 (6.86)	3.59 (7.89)	2.88 (5.36)	0.024*	0.45
Sha-Cal Inv (−) Peak (deg)	−0.56 (7.42)	−5.74 (6.31)	−3.30 (8.71)	–3.51 (5.70)	0.037*	0.54
Sha-Cal Eve (+) Peak (deg)	6.72 (3.29)	5.90 (2.95)	6.89 (2.35)	6.39 (1.88)	0.557	0.20
Vertical Impact Peak (BW)	1.13 (0.39)	1.14 (0.55)	1.21 (0.44)	1.09 (0.58)	0.129	0.37
Vertical Average Loading Rate (BW*s^−1^)	75.19 (46.43)	77.17 (57.52)	73.48 (43.17)	72.84 (65.73)	0.537	0.08
Peak Breaking Force (BW)	−0.24 (0.05)	−0.24 (0.07)	−0.24 (0.05)	−0.24 (0.05)	0.934	0.07

*Indicates significant differences.

MLA, medial longitudinal arch; ROM, range of motion; Inv, inversion; Eve, eversion; BW, bodyweight; Sha-Cal, calcaneus with respect to the shank joint angles.

Despite the IG being 2.42 times less likely to experience an RRI within the 1-year study period following the foot-core intervention (Taddei et al., 2020b), no reduction in loading rates—which are considered a biomechanical-related risk factor (Hreljac et al., 1999; Bredeweg et al., 2013; Davis et al., 2016; Napier et al., 2018)—was observed in the IG after 8 weeks (Table 3). In the present study, although changes in running biomechanics were assessed after the 8 weeks of intervention, the RRI incidence was calculated at 1 year. It is possible that the kinematic- and kinetic-related risk factors changed in the intervening time; however, we did not assess running biomechanics at 1 year, but only at 8 weeks. Future studies should further evaluate the effects of specific foot-ankle intervention strategies on the modification of the loading variables associated with RRIs throughout the full trial period and their relationship with the reduction of RRI risk.

The MLA range of motion (ROM) (*p* = 0.024) was significantly lower in the IG than in the CG at week 8 with a small effect size (Table 3). In a previous publication, we showed that the foot-core training strengthened some of the intrinsic foot muscles (abductor hallucis, flexor digitorum brevis, abductor digiti minimi, and flexor hallucis brevis) (Taddei et al., 2020a) responsible for sustaining the MLA (Fiolkowski et al., 2003), possibly increasing the resistance to its deformation during running and thus resulting in a smaller amount of arch collapse in the IG. This is consistent with what was reported by Mulligan and Cook (Mulligan and Cook, 2013), who found a decreased navicular drop after 4 weeks of intrinsic foot muscle training. The MLA should have the capacity to be flexible in response to running loads, allowing foot-joint adjustments to dampen impacts through multiple mechanisms, including stiffness and power absorption, but it must also be rigid enough to allow propulsion in the push-off phase (McDonald et al., 2016). Our foot-core training may have increased the ability of the plantar intrinsic muscles to provide force-dependent alterations in the MLA stiffness and to facilitate efficient foot-to-ground contact during running (Kelly, 2015; Kelly et al., 2018). An actively restricted MLA may help to decrease the mechanical demand on the soft tissues of the foot, such as ligaments, fascia, and tendons, and may result in fewer injuries in these structures, such as plantar fasciitis, which derives from repetitive abnormal strain and loading of the plantar fascia and flattening of the MLA (Wearing et al., 2006; Chang et al., 2014). A further mediation analysis could reveal if the changes observed in MLA behavior in the IG are associated with the reduction of RRI in our RCT (Taddei et al., 2020b). Further research should be conducted to determine how the changes in MLA pattern observed after the training program modify the running performance, because our previous proof-of-concept study showed that the foot-core training increased the vertical impulse during running (Taddei et al., 2020a).

The 8-week foot-core training affected the rearfoot inversion peak [shank-calcaneus (Sha-Cal) angle] as the rearfoot presented with increased inversion to the shank with respect to what was observed in controls with a medium effect size (*p* = 0.037; Table 3). We speculate that this is a consequence of the strengthening of the extrinsic foot-ankle muscles, such as the tibialis posterior, thus promoting the inversion of the calcaneus, resisting eversion during stance phase (Ivanenko et al., 2002; Neptune et al., 2008; Dubbeldam et al., 2013), and stabilizing the MLA (Imhauser et al., 2004; Kelly et al., 2015). The pathomechanics of medial tibial stress syndrome caused by periosteal inflammation is probably linked to excessive fascial traction caused by muscle tension resulting from excessive and/or prolonged pronation. A more inverted calcaneus in the IG may have increased the twisting of the osteoligamentous plate at initial ground contact (Araújo et al., 2019), which could consequently increase the resistance to pronation during the loading phase of running, when this plate tends to untwist. This increased resistance to calcaneus pronation in the IG may have provided the necessary protection for the tibiotalar joint from high traction forces imposed by the evertor and invertor muscles during the stance phase (Lundberg, 1989), resulting in less chance for an injury to occur in the IG than in the CG, whose members presented with more lower-leg RRI (Taddei et al., 2020b).

### The Effect of the Foot-Core Training on Foot-Ankle Kinematic Patterns During the Whole Stance Phase of Running.

In order to better describe and measure the complexity of the interaction between foot joints in running, we next explored changes resulting from the intervention in the 24 kinematic time series from the Rizzoli foot model (Phinyomark et al., 2018). We performed a vector analysis of the resultant angles using 1D-SPM to compare the CG and IG (Supplementary Figures). This approach does not rely on the experimenter’s subjective selection of the appropriate discrete variables, allowing changes to be identified in the whole time series that may have been missed using a discrete-parameter approach.

**FIGURE 2:**
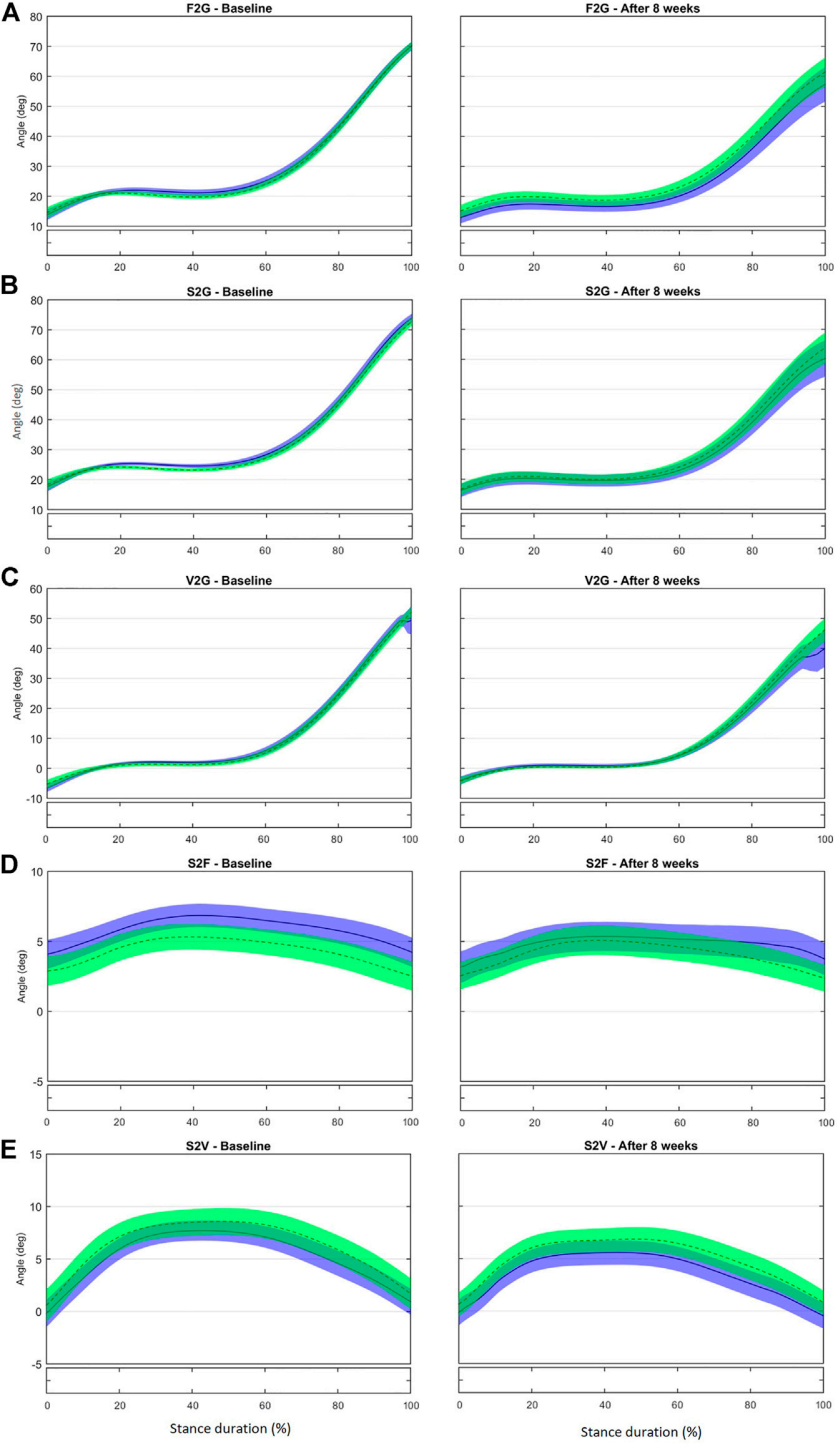
Mean (±1SD) joint rotation angles during normalized stance phase duration of running in the baseline (left) and after 8 weeks of intervention (right). From top to bottom: Sagittal-plane inclination of the first metatarsal bone to the ground **(A)**, of the second metatarsal bone to the ground **(B)**, and of the fifth metatarsal bone to the ground **(C)**; transverse-plane divergence between first and second metatarsal bones **(D)** and between fifth and second metatarsal bones **(E)**. Green, CG group; Blue, IG group. The black bar below the graph represents the time during which the differences between the groups occurred (*p* < 0.05), what was indicated by the SPM{t} statistics.

Sagittal-plane inclination of the metatarsal bones to the ground [first metatarsal bone to the ground (F2G), second metatarsal bone to the ground (S2G), and fifth metatarsal bone to the ground (V2G)] and metatarsal bone divergence in the transverse plane of the foot [second to first (S2F) and second to fifth (S2V)] were not different between the IG and CG after 8 weeks (Figure 2, Supplementary Figures S1–3). Although our previous proof-of-concept study (Taddei et al., 2020a) showed that the foot-core training increased the muscle volume of the abductor digiti minimi and flexor digitorum brevis, the full RCT did not result in changes in the kinematics of the metatarsal bones. We had expected an increase of F2G, S2G, and V2G as the MLA raised and shortened, but these changes may be very small and difficult to detect with skin markers.

**FIGURE 3:**
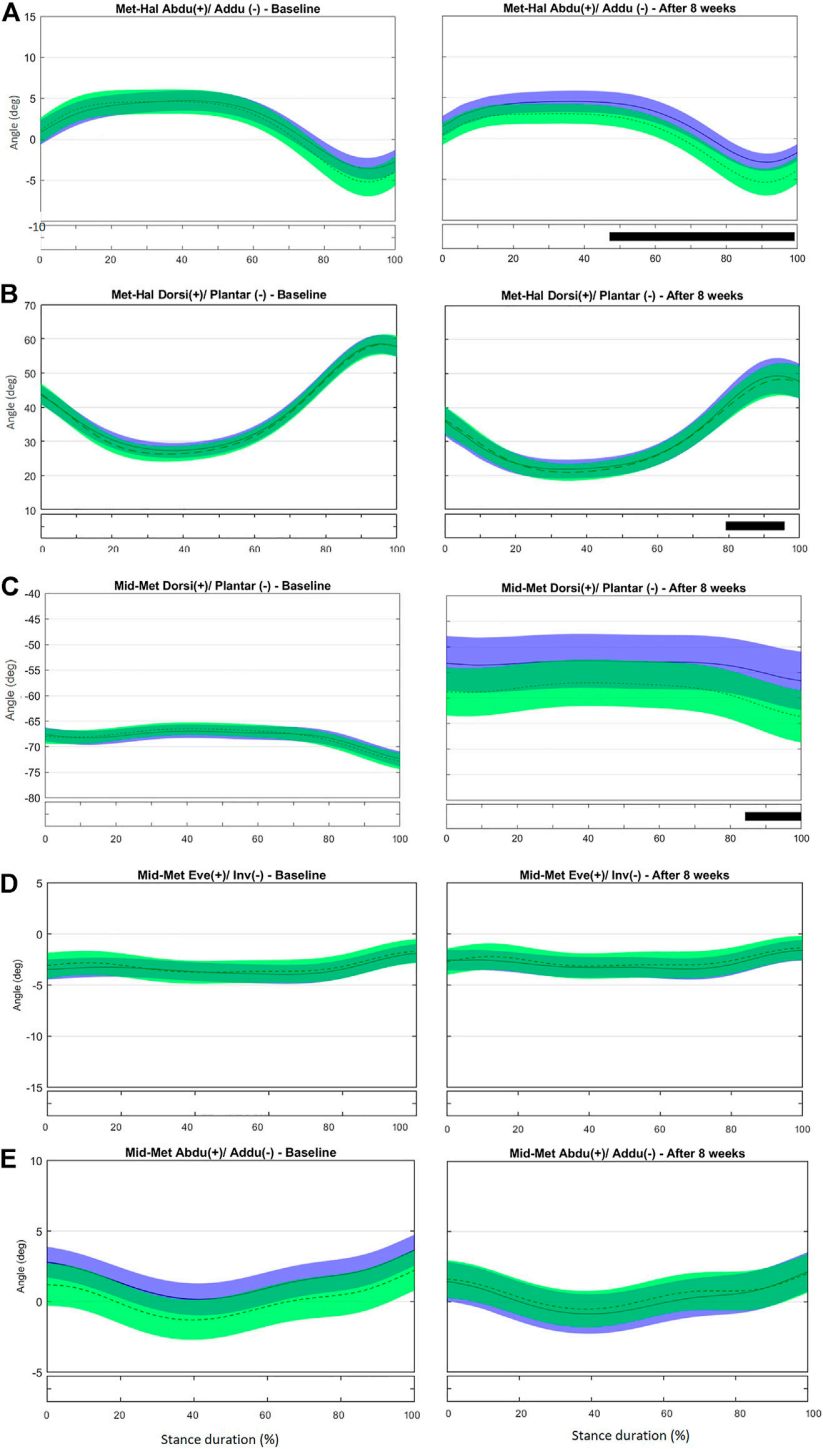
Mean (±1SD) joint rotation angles during normalized stance phase duration of running in the baseline (left) and after 8 weeks of intervention (right). From top to bottom: transverse-plane rotations between hallux and metatarsus **(A)**; sagittal-plane rotations between hallux and metatarsus **(B)**; sagittal-plane rotations between metatarsus and midfoot **(C)**, frontal-plane rotations between metatarsus and midfoot **(D)**, and transverse-plane rotations between metatarsus and midfoot **(E)**. Green, CG group; Blue, IG group. The black bar below the graph represents the time during which the differences between the groups occurred (*p* < 0.05), what was indicated by the SPM{t} statistics.

No difference was observed in the kinematics of the first metatarsus-phalangeal (MTP) joint (Met-Hal) between the groups at baseline (Figures 3A,B and Supplementary Figure S4). After the intervention, the IG presented with increased abduction in the period from 47 to 99% of stance; the first MTP joint retuned to a less adducted (t*2.702, *p* = 0.003) and more dorsiflexed position (t*2.783, *p* = 0.003) at push-off (79–96%) compared with controls (Figures 3A,B). Greater hallux abduction may be the consequence of a stronger abductor hallucis due to the intervention. This muscle also acts as a dynamic elevator of the MLA by increasing the tension in the plantar fascia that connects the MTP joint and calcaneus (the windlass mechanism) (Wong, 2007; Caravaggi et al., 2009; Jung et al., 2011a; McDonald et al., 2016). Thus, this greater hallux abduction may also explain the resulting reduction in MLA ROM found in the discrete analysis.

**FIGURE 4:**
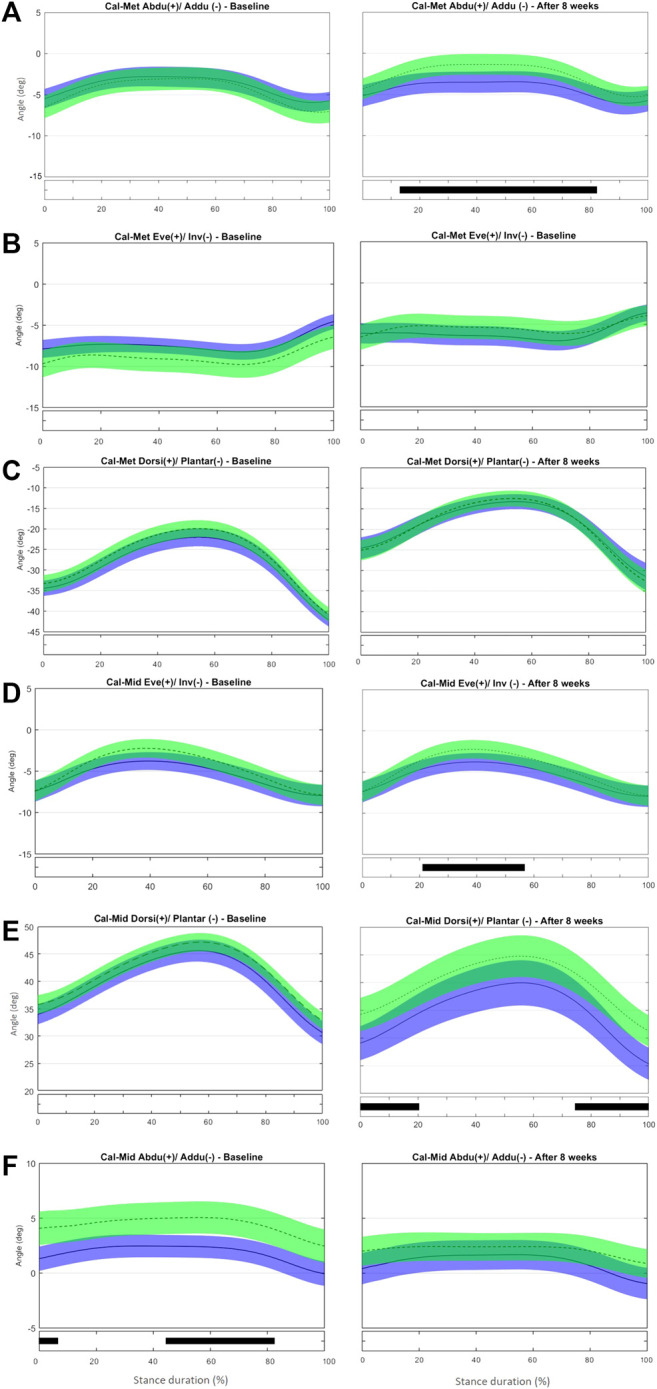
Mean (±1SD) joint rotation angles during normalized stance phase duration of running in the baseline (left) and after 8 weeks of intervention (right). From top to bottom: transverse-plane rotations between metatarsus and calcaneus **(A)**, frontal-plane rotations between metatarsus and calcaneus **(B)**, sagittal-plane rotations between metatarsus and calcaneus **(C)**, frontal-plane rotations between midfoot and calcaneus **(D)**, sagittal-plane rotations between midfoot and calcaneus **(E)**, and transverse-plane rotations midfoot and calcaneus **(F)**. Green, CG group; Blue, IG group. The black bar below the graph represents the time during which the differences between the groups occurred (*p* < 0.05), what was indicated by the SPM{t} statistics.

No difference was observed in midfoot-metatarsus (Mid-Met) (Figures 3C–E) and calcaneus-metatarsus (Cal-Met) (Figures 4A–C) kinematics between the IG and CG at baseline. The intervention had the effect in the sagittal-plane motion of Mid-Met of reducing metatarsal bone plantarflexion from 84 to 100% of stance compared with controls (t*2.764, *p* = 0.016) (Figure 3C and Supplementary Figure S5). After the foot-core training, the reduction in the Mid-Met plantarflexion toward a dorsiflexion from 84% to push-off may be a consequence of a more fixed position of the midfoot and metatarsal bones relative to the ground at push-off. The position of the midfoot (Cal-Mid) at push-off may have influenced the metatarsal segment (Mid-Met) as in a closed kinetic chain, leading this segment to move in the same direction as the midfoot (Takabayashi et al., 2018), and thus resulting in more dorsiflexion of the first metatarsus-hallux joint (Met-Hal), as discussed previously. After the intervention, Cal-Met adduction increased in the IG relative to the CG from 13 to 82% of stance (t*2.722, *p* = 0.008) (Figure 4A and Supplementary Figure S6). The concomitant reduction in MLA ROM (discrete analysis) seems to show that the IG developed a “stiffer” foot that behaves like a rigid lever, allowing greater plantarflexion torque to be transmitted to the ground during running (Donatelli, 1985). Further research is needed to determine how these changes in the MLA and Cal-Met patterns affect the running performance. Although Messier et al. (2018) did not find differences in forefoot adduction between injured and uninjured runners in a 2-year prospective study, the present foot-core training changed the metatarsal position and motion during running; a mediation effect analysis should be performed to assess how this change was related to the lower RRI incidence observed in the IG (Taddei et al., 2020b).

At baseline, no difference was observed in frontal- and sagittal-plane midfoot-to-calcaneus angles (Cal-Mid) between the IG and CG. After the training program, the CG showed lower Cal-Mid inversion from 25 to 45% of stance (t*2.704, *p* = 0.014) (Figure 4D and Supplementary Figure S7). The IG presented a reduced Cal-Mid dorsiflexion at early stance (0–20% of stance; t*2.820, *p* = 0.014) and at push-off (80–100% of stance; t*2.820, *p* = 0.013) compared with controls after 8 weeks (Figure 4E). The CG showed a less-abducted Cal-Mid at baseline from 0–0.7% and 44–80% of stance (t*2.630, *p* = 0.017 and *p* = 0.015, respectively). After 8 weeks of training, there was no difference between IG and CG (Figure 4F).

**FIGURE 5:**
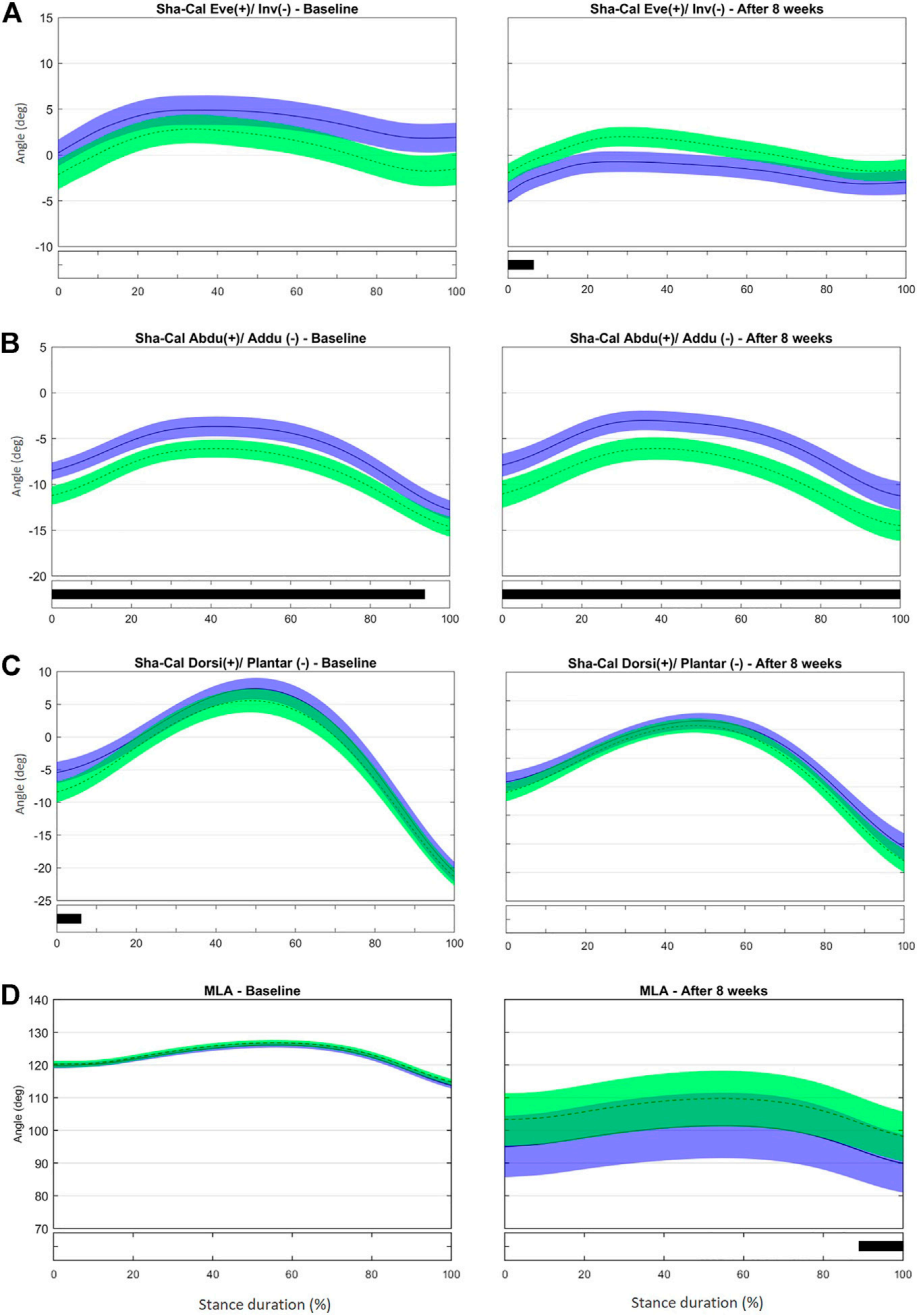
Mean (±1SD) joint rotation angles during normalized stance phase duration of running in the baseline (left) and after 8 weeks of intervention (right). From top to bottom, frontal-plane rotations between calcaneus and shank **(A)**, transverse-plane rotations between calcaneus and shank **(B)**, sagittal-plane rotations between calcaneus and shank **(C)**, and medial longitudinal arch angle **(D)**. Green, CG group; Blue, IG group. The black bar below the graph represents the time during which the differences between the groups occurred (*p* < 0.05), what was indicated by the SPM{t} statistics.

At baseline, no difference between groups was observed in the frontal-plane Sha-Cal. At the 8-week assessment, calcaneus inversion at initial contact (0–6% stance, t* = 1.969, *p* = 0.05, Supplementary Figure S8) was greater in the IG than in the CG (Figure 5A). This result is consistent with the outcome of the discrete analysis that showed a more inverted rearfoot in the IG. As stated before, a more inverted calcaneus at early stance may help to attenuate the impact forces to the tibiotalar joint and diminish tibia rotation (Deschamps et al., 2019), and thus may have contributed to the reduced RRI incidence in the IG (Bouché and Johnson, 2007).

At baseline, the IG showed a less adducted Sha-Cal than the CG for most of stance duration (0–94% stance, t*2.694, *p* < 0.001), and a significant change was observed after 8 weeks (0–100% stance, t*2.788, *p* < 0.001) (Figure 5B and Supplementary Figure S9). Because the groups were different for most of the stance duration at baseline and maintained those differences after 8 weeks, the intervention does not seem to be responsible for the changes in the transverse Sha-Cal pattern observed in the IG after the training. No difference was found in the Sha-Cal sagittal-plane angle between groups after the intervention (Figure 5C).

Different from what we found in the discrete analysis; the SPM analysis did not reveal differences between the groups in the MLA excursion during any part of the stance phase after 8 weeks (Figure 5D and Supplementary Figure S3B]. Both groups presented with more pronounced MLA angles, with large variability in the ROM within each group at late stance, after 8 weeks than at baseline (89–100% stance; t* = 2.521, *p* = 0.025). In addition, both the IG and CG had significantly greater variability in the MLA pattern after 8 weeks. Both protocols (intervention and control) may have affected MLA biomechanics, albeit in different directions, thus making it difficult to determine the differences between the groups. It is notable that a stretching protocol designed as a placebo intervention affected MLA kinematics. We chose a simple stretching protocol as the control because most participants were part of running groups that already had some sort of stretching routine. Because most participants would combine their running practice with muscle stretching, the CG adherence to the protocol was not tracked thoroughly. The stretching exercises resulted in stretching of the Achilles tendon and foot-ankle plantar flexor muscles that may directly influence the calcaneus inclination; this could in turn modify the tension in the plantar fascia and, consequently, the passive support of the MLA (Yeap et al., 2001; DiGiovanni et al., 2003). Thus, the potential changes in the MLA pattern expressed by the increased variability in the CG may be a consequence of the changes in plantar fascia tension and calcaneus position and motion enhanced by the variability in the amount of stretching and also in individual response to the movement. However, another explanation may be considered. According to Pataky et al. (2013), in some cases scalar extraction analysis, based on the extraction of discrete variables that appear to have maximum effect, reaches significance and SPM analysis does not. Comparing discrete variables means, in fact, considering the comparison of only one sample of the entire time series and discarding the remaining samples for the comparisons.

In summary, the IG runners had decreased medial longitudinal arch excursion and increased rearfoot inversion. After the intervention, recreational runners also landed with a more inverted calcaneus in relation to the shank, and a less dorsiflexed midfoot with respect to the calcaneus, than did controls. At midstance, the metatarsus was less plantarflexed relative to the midfoot and more adducted relative to the calcaneus, and the first MTP joint was more abducted in the IG than in the controls. Last, the intervention resulted in a less dorsiflexed midfoot to the calcaneus at push-off, and in a less adducted and more dorsiflexed MTP joint.

### Strengths and Limitations

The strengths of our study are the rigorous method for the RCT, its high completion and small dropout rates at follow-up, the adoption of robust statistical models (GLMM and 1D-SPM) that consider the complex non-linear iterations of foot-joint biomechanics, and its large sample size compared with other studies in the same field (Jung et al., 2011b; Goldmann et al., 2013; Baltich et al., 2015; Campitelli et al., 2016; Lucas-Cuevas et al., 2016; Mølgaard et al., 2018).

This study also had some limitations. First, we did not assess running biomechanics at the 1-year follow-up as we did for RRI incidence; thus, we could not draw any conclusions about the causality between RRI incidence and kinetic- or kinematic-related risk factors for RRI affected by our training program. Second, although different RRIs or injury sites are expected to originate from different mechanisms, and enhancing foot strength might be more effective in preventing some types of injuries than others (some of them reduced impact loading, some did not, thus the mean of the group did not show between-group differences), we could not differentiate between different types of RRIs. It is important to note that the most serious RRIs were in runners from CG—stress fractures, which could also be related to the difference in the impact damping mechanisms performed by the musculoskeletal system, more specifically the foot-core, that was not strengthened as in the IG, where those type of RRIs did not occur.

This prevented us from explaining the biomechanical mechanisms for the reduction of RRI incidence in the IG after the intervention. Furthermore, although lower-limb kinematic patterns are similar in over-ground and treadmill running (Fellin et al., 2010; Sinclair et al., 2014), the participants ran barefoot on a treadmill to facilitate kinematic measurements, a condition different from their usual practice. Finally, we observed some differences at baseline in the foot-ankle kinematic patterns that could be related to the previously identified clusters of movement patterns among our population of recreational runners included in this study (Watari et al., 2021). Runners in the different clusters might have responded differently to the training program. If true, this would suggest that the response to the exercise intervention depends on the individual foot biomechanical pattern, which could explain the absence of differences in some discrete and continuous outcomes analyzed.

## Conclusion

The 8-week foot-core exercise program significantly changed the kinematic patterns of the ankle, tarso-metarsal, midtarsal, and MPT joints and some of the biomechanical risk factors for RRI, such as MLA ROM and rearfoot angle. No effect was observed on impact and breaking forces or on loading rates. After the intervention, recreational runners landed with a significantly less dorsiflexed midfoot, and a more inverted calcaneus compared to controls. At midstance, runners run with a significantly more abducted hallux, a less plantarflexed and more adducted forefoot. And finally, the intervention resulted in a push-off with a significantly less dorsiflexed midfoot, a less adducted and more plantarflexed hallux. Although a further mediation analysis should be performed, the observed changes in foot-joint kinematics may be responsible for the reduction in RRI incidence following the foot-core training program.

## Data Availability

The original contributions presented in the study are publicly available. This data can be found here: https://figshare.com/search?q=10.6084%2Fm9.figshare.13484715.

## References

[B1] Araújo V. L. Souza T. R. Magalhães F. A. Santos T. R. T. Holt K. G. Fonseca S. T. (2019). Effects of a Foot Orthosis Inspired by the Concept of a Twisted Osteoligamentous Plate on the Kinematics of Foot-Ankle Complex during Walking: A Proof of Concept. J. Biomech. 93, 118–125. 10.1016/j.jbiomech.2019.06.020 31288932

[B2] Baltich J. Emery C. A. Whittaker J. L. Nigg B. M. (2017). Running Injuries in Novice Runners Enrolled in Different Training Interventions: a Pilot Randomized Controlled Trial. Scand. J. Med. Sci. Sports 27, 1372–1383. 10.1111/sms.12743 27486011

[B3] Baltich J. Maurer C. Nigg B. M. (2015). Increased Vertical Impact Forces and Altered Running Mechanics with Softer Midsole Shoes. PLoS One 10, e0125196–11. 10.1371/journal.pone.0125196 25897963 PMC4405580

[B4] Bennell K. L. Malcolm S. A. Thomas S. A. Wark J. D. Brukner P. D. (1996). The Incidence and Distribution of Stress Fractures in Competitive Track and Field Athletes. Am. J. Sports Med. 24, 211–217. 10.1177/036354659602400217 8775123

[B5] Bennett J. E. Reinking M. F. Pluemer B. Pentel A. Seaton M. Killian C. (2001). Factors Contributing to the Development of Medial Tibial Stress Syndrome in High School Runners. J. Orthop. Sports Phys. Ther. 31, 504–510. 10.2519/jospt.2001.31.9.504 11570734

[B6] Bouche R. T. Johnson C. H. (2007). Medial Tibial Stress Syndrome (Tibial Fasciitis). J. Am. Podiatr. Med. Assoc. 97, 31–36. 10.7547/0970031 17218623

[B7] Bredeweg S. W. Kluitenberg B. Bessem B. Buist I. (2013). Differences in Kinetic Variables between Injured and Noninjured Novice Runners: A Prospective Cohort Study. J. Sci. Med. Sport 16, 205–210. 10.1016/j.jsams.2012.08.002 22921763

[B8] Brumitt J. (2009). Injury Prevention for High School Female Cross-Country Athletes. Athl. Ther. Today 14, 8–12. 10.1123/att.14.4.8

[B9] Buist I. Bredeweg S. W. Bessem B. van Mechelen W. Lemmink K. A. P. M. Diercks R. L. (2010). Incidence and Risk Factors of Running-Related Injuries during Preparation for a 4-mile Recreational Running Event. Br. J. Sports Med. 44, 598–604. 10.1136/bjsm.2007.044677 18487252

[B10] Buist I. Bredeweg S. W. van Mechelen W. Lemmink K. A. P. M. Pepping G.-J. Diercks R. L. (2008). No Effect of a Graded Training Program on the Number of Running-Related Injuries in Novice Runners. Am. J. Sports Med. 36, 33–39. 10.1177/0363546507307505 17940147

[B11] Busseuil C. Freychat P. Guedj E. B. Lacour J. R. (1998). Rearfoot-forefoot Orientation and Traumatic Risk for Runners. Foot Ankle Int. 19, 32–37. 10.1177/107110079801900106 9462910

[B12] Campitelli N. A. Spencer S. A. Bernhard K. Heard K. Kidon A. (2016). Effect of Vibram Fivefingers Minimalist Shoes on the Abductor Hallucis Muscle. J. Am. Podiatr. Med. Assoc. 106, 344–351. 10.7547/14-084 27762613

[B13] Caravaggi P. Matias A. B. Taddei U. T. Ortolani M. Leardini A. Sacco I. C. N. (2019). Reliability of Medial-Longitudinal-Arch Measures for Skin-Markers Based Kinematic Analysis. J. Biomech. 88, 180–185. 10.1016/j.jbiomech.2019.03.017 30910360

[B14] Caravaggi P. Pataky T. Goulermas J. Y. Savage R. Crompton R. (2009). A Dynamic Model of the Windlass Mechanism of the Foot: Evidence for Early Stance Phase Preloading of the Plantar Aponeurosis. J. Exp. Biol. 212, 2491–2499. 10.1242/jeb.025767 19617443

[B15] Ceyssens L. Vanelderen R. Barton C. Malliaras P. Dingenen B. (2019). Biomechanical Risk Factors Associated with Running-Related Injuries: A Systematic Review. Sports Med. 49, 1095–1115. 10.1007/s40279-019-01110-z 31028658

[B16] Chang R. Rodrigues P. A. Van Emmerik R. E. A. Hamill J. (2014). Multi-segment Foot Kinematics and Ground Reaction Forces during Gait of Individuals with Plantar Fasciitis. J. Biomech. 47, 2571–2577. 10.1016/j.jbiomech.2014.06.003 24992816

[B17] Cohen J. (1988). in Statistical Power Analysis for the Behavioral Sciences. Editor Associates L. E. (Academic Press).

[B18] Cowan D. N. Jones B. H. Robinson J. R. (1993). Foot Morphologic Characteristics and Risk of Exercise-Related Injury. Arch. Fam. Med. 2, 773–777. 10.1001/archfami.2.7.773 7906597

[B19] Davis I. S. Bowser B. J. Mullineaux D. R. (2016). Greater Vertical Impact Loading in Female Runners with Medically Diagnosed Injuries: A Prospective Investigation. Br. J. Sports Med. 50, 887–892. 10.1136/bjsports-2015-094579 26644428

[B20] Davis I. S. Rice H. M. Wearing S. C. (2017). Why Forefoot Striking in Minimal Shoes Might Positively Change the Course of Running Injuries. J. Sport Health Sci. 6, 154–161. 10.1016/j.jshs.2017.03.013 30356630 PMC6189002

[B21] Deschamps K. Eerdekens M. Peters H. Matricali G. A. Staes F. (2019). Multi-segment Foot Kinematics during Running and its Association with Striking Patterns. Sports Biomech. 21, 71–84. 10.1080/14763141.2019.1645203 31464161

[B22] DiGiovanni B. F. Nawoczenski D. A. Lintal M. E. Moore E. A. Murray J. C. Wilding G. E. (2003). Tissue-specific Plantar Fascia-Stretching Exercise Enhances Outcomes in Patients with Chronic Heel Pain. The J. Bone Jt. Surgery-American Volume 85, 1270–1277. 10.2106/00004623-200307000-00013 12851352

[B23] Donatelli R. (1985). Normal Biomechanics of the Foot and Ankle. J. Orthop. Sports Phys. Ther. 7, 91–95. 10.2519/jospt.1985.7.3.91 18802279

[B24] Dubbeldam R. Nester C. Nene A. V. Hermens H. J. Buurke J. H. (2013). Kinematic Coupling Relationships Exist between Non-adjacent Segments of the Foot and Ankle of Healthy Subjects. Gait & Posture 37, 159–164. 10.1016/j.gaitpost.2012.06.033 22951211

[B25] Dudley R. I. Pamukoff D. N. Lynn S. K. Kersey R. D. Noffal G. J. (2017). A Prospective Comparison of Lower Extremity Kinematics and Kinetics between Injured and Non-injured Collegiate Cross Country Runners. Hum. Mov. Sci. 52, 197–202. 10.1016/j.humov.2017.02.007 28237655

[B26] Farris D. J. Kelly L. A. Cresswell A. G. Lichtwark G. A. (2019). The Functional Importance of Human Foot Muscles for Bipedal Locomotion. Proc. Natl. Acad. Sci. U.S.A. 116, 1645–1650. 10.1073/pnas.1812820116 30655349 PMC6358692

[B27] Fellin R. E. Manal K. Davis I. S. (2010). Comparison of Lower Extremity Kinematic Curves during Overground and Treadmill Running. J. Appl. Biomech. 26, 407–414. 10.1126/scisignal.2001449.Engineering10.1123/jab.26.4.407 21245500 PMC3266869

[B28] Feltner M. E. MacRae H. S. Macrae P. G. Turner N. S. Hartman C. A. Summers M. L. (1994). Strength Training Effects on Rearfoot Motion in Running. Med. Sci. Sports Exerc. 26, 1021–1027. 10.1249/00005768-199408000-00014 7968419

[B29] Fiolkowski P. Brunt D. Bishop M. Woo R. Horodyski M. (2003). Intrinsic Pedal Musculature Support of the Medial Longitudinal Arch: an Electromyography Study. J. Foot Ankle Surg. 42, 327–333. 10.1053/j.jfas.2003.10.003 14688773

[B30] Fokkema T. Vos R.-J. d. Van Ochten J. M. van Ochten J. A. Davis I. S. Bindels P. J. (2017). Preventing Running-Related Injuries Using Evidence-Based Online Advice: The Design of a Randomised-Controlled Trial. BMJ Open Sport Exerc. Med. 3, e000265–9. 10.1136/bmjsem-2017-000265 PMC5530119 28761721

[B31] Fredericson M. Moore T. (2005). Muscular Balance, Core Stability, and Injury Prevention for Middle- and Long-Distance Runners. Phys. Med. Rehabil. Clin. North America 16, 669–689. 10.1016/j.pmr.2005.03.001 16005399

[B32] Goldmann J.-P. Sanno M. Willwacher S. Heinrich K. Brüggemann G.-P. (2013). The Potential of Toe Flexor Muscles to Enhance Performance. J. Sports Sci. 31, 424–433. 10.1080/02640414.2012.736627 23106289

[B33] Grimston S. K. Engsberg J. R. Kloiber R. Hanley D. A. (1991). Bone Mass, External Loads, and Stress Fracture in Female Runners. Int. J. Sport Biomech. 7, 293–302. 10.1123/ijsb.7.3.293

[B34] Grood E. S. Suntay W. J. (1983). A Joint Coordinate System for the Clinical Description of Three-Dimensional Motions: Application to the Knee. J. Biomech. Eng. 105, 136–144. 10.1115/1.3138397 6865355

[B35] Headlee D. L. Leonard J. L. Hart J. M. Ingersoll C. D. Hertel J. (2008). Fatigue of the Plantar Intrinsic Foot Muscles Increases Navicular Drop. J. Electromyogr. Kinesiol. 18, 420–425. 10.1016/j.jelekin.2006.11.004 17208458

[B36] Hein T. Grau S. (2014). Can Minimal Running Shoes Imitate Barefoot Heel-Toe Running Patterns? A Comparison of Lower Leg Kinematics. J. Sport Health Sci. 3, 67–73. 10.1016/j.jshs.2014.03.002

[B37] Hespanhol L. C. van Mechelen W. Verhagen E. (2018). Effectiveness of Online Tailored Advice to Prevent Running-Related Injuries and Promote Preventive Behaviour in Dutch Trail Runners: a Pragmatic Randomised Controlled Trial. Br. J. Sports Med. 52, 851–858. 10.1136/bjsports-2016-097025 28855183

[B38] Hollman J. H. Kolbeck K. E. Hitchcock J. L. Koverman J. W. Krause D. A. (2006). Correlations between Hip Strength and Static Foot and Knee Posture. J. Sport Rehabil. 15, 12–23. 10.1123/jsr.15.1.12

[B39] Hott A. Liavaag S. Juel N. G. Brox J. I. (2015). Study Protocol: A Randomised Controlled Trial Comparing the Long Term Effects of Isolated Hip Strengthening, Quadriceps-Based Training and Free Physical Activity for Patellofemoral Pain Syndrome (Anterior Knee Pain). BMC Musculoskelet. Disord. 16, 1–8. 10.1186/s12891-015-0493-6 25879452 PMC4342827

[B40] Hreljac A. Marshall R. N. Hume P. A. (1999). Evaluation of Lower Extremity Overuse Injury Potential in Runners. Med. Sci. Sports Exerc. 32, 1635–1641. 10.1097/00005768-200009000-00018 10994917

[B41] Hreljac A. Marshall R. N. Hume P. A. (2000). Evaluation of Lower Extremity Overuse Injury Potential in Runners. Med. Sci. Sports Exerc. 32, 1635–1641. 10.1097/00005768-200009000-00018 10994917

[B42] Imhauser C. W. Siegler S. Abidi N. A. Frankel D. Z. (2004). The Effect of Posterior Tibialis Tendon Dysfunction on the Plantar Pressure Characteristics and the Kinematics of the Arch and the Hindfoot. Clin. Biomech. 19, 161–169. 10.1016/j.clinbiomech.2003.10.007 14967579

[B43] Ivanenko Y. P. Grasso R. Macellari V. Lacquaniti F. (2002). Control of Foot Trajectory in Human Locomotion: Role of Ground Contact Forces in Simulated Reduced Gravity. J. Neurophysiol. 87, 3070–3089. 10.1152/jn.2002.87.6.3070 12037209

[B44] Johnston C. A. Taunton J. E. Lloyd-Smith D. R. McKenzie D. C. (2003). Preventing Running Injuries. Practical Approach for Family Doctors. Can. Fam. Physician 49, 1101–1109. 14526862 PMC2214294

[B45] Jung D.-Y. Kim M.-H. Koh E.-K. Kwon O.-Y. Cynn H.-S. Lee W.-H. (2011a). A Comparison in the Muscle Activity of the Abductor Hallucis and the Medial Longitudinal Arch Angle during Toe Curl and Short Foot Exercises. Phys. Ther. Sport 12, 30–35. 10.1016/j.ptsp.2010.08.001 21256447

[B46] Jung D.-Y. Koh E.-K. Kwon O.-Y. (2011b). Effect of Foot Orthoses and Short-Foot Exercise on the Cross-Sectional Area of the Abductor Hallucis Muscle in Subjects with Pes Planus: A Randomized Controlled Trial1. Bmr 24, 225–231. 10.3233/BMR-2011-0299 22142711

[B47] Kelly L. A. Cresswell A. G. Farris D. J. (2018). The Energetic Behaviour of the Human Foot across a Range of Running Speeds. Sci. Rep. 8, 10576. 10.1038/s41598-018-28946-1 30002498 PMC6043578

[B48] Kelly L. A. Lichtwark G. Cresswell A. G. Cresswell A. G. (2015). Active Regulation of Longitudinal Arch Compression and Recoil during Walking and Running. J. R. Soc. Interf. 12, 20141076. 10.1098/rsif.2014.1076 PMC4277100 25551151

[B49] Kelly L. (2015). *In-vivo* Function of Human Plantar Intrinsic Foot Muscles. 10.14264/uql.2015.341

[B50] Ker R. F. Bennett M. B. Bibby S. R. Kester R. C. Alexander R. M. (1987). The spring in the Arch of the Human Foot. Nature 325, 147–149. 10.1038/325147a0 3808070

[B51] Kluitenberg B. van Middelkoop M. Diercks R. van der Worp H. (2015). What Are the Differences in Injury Proportions between Different Populations of Runners? A Systematic Review and Meta-Analysis. Sports Med. 45, 1143–1161. 10.1007/s40279-015-0331-x 25851584 PMC4513221

[B52] Kuhman D. J. Paquette M. R. Peel S. A. Melcher D. A. (2016). Comparison of Ankle Kinematics and Ground Reaction Forces between Prospectively Injured and Uninjured Collegiate Cross Country Runners. Hum. Mov. Sci. 47, 9–15. 10.1016/j.humov.2016.01.013 26827155

[B53] Leardini A. Benedetti M. G. Berti L. Bettinelli D. Nativo R. Giannini S. (2007). Rear-foot, Mid-foot and Fore-Foot Motion during the Stance Phase of Gait. Gait & Posture 25, 453–462. 10.1016/j.gaitpost.2006.05.017 16965916

[B54] Lucas-Cuevas A. G. Baltich J. Enders H. Nigg S. Nigg B. (2016). Ankle Muscle Strength Influence on Muscle Activation during Dynamic and Static Ankle Training Modalities. J. Sports Sci. 34, 803–810. 10.1080/02640414.2015.1072640 26228260

[B55] Lundberg A. (1989). Kinematics of the Ankle and Foot: *In Vivo* Roentgen Stereophotogrammetry. Acta Orthopaedica Scand. 60, 1–26. 10.3109/17453678909154185 PMC3323658 2686345

[B56] Matias A. B. Taddei U. T. Duarte M. Sacco I. C. N. (2016). Protocol for Evaluating the Effects of a Therapeutic Foot Exercise Program on Injury Incidence, Foot Functionality and Biomechanics in Long-Distance Runners: a Randomized Controlled Trial. BMC Musculoskelet. Disord. 17, 160. 10.1186/s12891-016-1016-9 27075480 PMC4831173

[B57] McDonald K. A. Stearne S. M. Alderson J. A. North I. Pires N. J. Rubenson J. (2016). The Role of Arch Compression and Metatarsophalangeal Joint Dynamics in Modulating Plantar Fascia Strain in Running. PLoS One 11, e0152602–16. 10.1371/journal.pone.0152602 27054319 PMC4824348

[B58] McKeon P. O. Fourchet F. (2015a). Freeing the Foot. Clin. Sports Med. 34 (2), 347–361. 10.1016/j.csm.2014.12.002 25818718

[B59] McKeon P. O. Hertel J. Bramble D. Davis I. (2015b). The Foot Core System: a New Paradigm for Understanding Intrinsic Foot Muscle Function. Br. J. Sports Med. 49, 290. 10.1136/bjsports-2013-092690 24659509

[B60] Messier S. P. Edwards D. G. Martin D. F. Lowery R. B. Cannon D. W. James M. K. (1995). Etiology of Iliotibial Band Friction Syndrome in Distance Runners. Med. Sci. Sports Exerc. 27, 951–960. 10.1249/00005768-199507000-00002 7564981

[B61] Messier S. P. Martin D. F. Mihalko S. L. Ip E. DeVita P. Cannon D. W. (2018). A 2-Year Prospective Cohort Study of Overuse Running Injuries: The Runners and Injury Longitudinal Study (TRAILS). Am. J. Sports Med. 46, 2211–2221. 10.1177/0363546518773755 29791183

[B62] Messier S. P. Pittala K. A. (1988). Etiologic Factors Associated with Selected Running Injuries. Med. Sci. Sports Exerc. 20, 501–505. 10.1249/00005768-198810000-00012 3193867

[B63] Milner C. E. Ferber R. Pollard C. D. Hamill J. Davis I. S. (2006). Biomechanical Factors Associated with Tibial Stress Fracture in Female Runners. Med. Sci. Sports Exerc. 38, 323–328. 10.1249/01.mss.0000183477.75808.92 16531902

[B64] Mølgaard C. M. Rathleff M. S. Andreasen J. Christensen M. Lundbye-Christensen S. Simonsen O. (2018). Foot Exercises and Foot Orthoses Are More Effective Than Knee Focused Exercises in Individuals with Patellofemoral Pain. J. Sci. Med. Sport 21, 10–15. 10.1016/j.jsams.2017.05.019 28844333

[B65] Mulligan E. P. Cook P. G. (2013). Effect of Plantar Intrinsic Muscle Training on Medial Longitudinal Arch Morphology and Dynamic Function. Man. Ther. 18, 425–430. 10.1016/j.math.2013.02.007 23632367

[B66] Napier C. MacLean C. L. Maurer J. Taunton J. E. Hunt M. A. (2018). Kinetic Risk Factors of Running-Related Injuries in Female Recreational Runners. Scand. J. Med. Sci. Sports 28, 2164–2172. 10.1111/sms.13228 29846979

[B67] Neptune R. R. Sasaki K. Kautz S. A. (2008). The Effect of Walking Speed on Muscle Function and Mechanical Energetics. Gait & Posture 28, 135–143. 10.1016/j.gaitpost.2007.11.004 18158246 PMC2409271

[B68] Nigg B. M. Baltich J. Federolf P. Manz S. Nigg S. (2017). Functional Relevance of the Small Muscles Crossing the Ankle Joint - the Bottom-Up Approach. Ciss 2. 10.15203/ciss_2017.003

[B69] Nigg B. M. Khan A. Fisher V. Stefanyshyn D. (1998). Effect of Shoe Insert Construction on Foot and Leg Movement. Med. Sci. Sports Exerc. 30, 550–555. 10.1097/00005768-199804000-00013 9565937

[B70] Noehren B. Davis I. Hamill J. (2007). ASB Clinical Biomechanics Award Winner 2006. Clin. Biomech. 22, 951–956. 10.1016/j.clinbiomech.2007.07.001 17728030

[B71] Noehren B. Hamill J. Davis I. (2013). Prospective Evidence for a Hip Etiology in Patellofemoral Pain. Med. Sci. Sports Exerc. 45, 1120–1124. 10.1249/MSS.0b013e31828249d2 23274607

[B72] Palmer K. Hebron C. Williams J. M. (2015). A Randomised Trial into the Effect of an Isolated Hip Abductor Strengthening Programme and a Functional Motor Control Programme on Knee Kinematics and Hip Muscle Strength. BMC Musculoskelet. Disord. 16, 1–8. 10.1186/s12891-015-0563-9 25935843 PMC4424529

[B73] Pataky T. C. Robinson M. A. Vanrenterghem J. (2018). A Computational Framework for Estimating Statistical Power and Planning Hypothesis-Driven Experiments Involving One-Dimensional Biomechanical Continua. J. Biomech. 66, 159–164. 10.1016/j.jbiomech.2017.09.031 29146283

[B74] Pataky T. C. Robinson M. A. Vanrenterghem J. (2013). Vector Field Statistical Analysis of Kinematic and Force Trajectories. J. Biomech. 46, 2394–2401. 10.1016/j.jbiomech.2013.07.031 23948374

[B75] Phinyomark A. Petri G. Ibáñez-Marcelo E. Osis S. T. Ferber R. (2018). Analysis of Big Data in Gait Biomechanics: Current Trends and Future Directions. J. Med. Biol. Eng. 38, 244–260. 10.1007/s40846-017-0297-2 29670502 PMC5897457

[B76] Pohl M. B. Buckley J. G. (2008). Changes in Foot and Shank Coupling Due to Alterations in Foot Strike Pattern during Running. Clin. Biomech. 23, 334–341. 10.1016/j.clinbiomech.2007.09.016 18006125

[B77] Portinaro N. Leardini A. Panou A. Monzani V. Caravaggi P. (2014). Modifying the Rizzoli Foot Model to Improve the Diagnosis of Pes-Planus: Application to Kinematics of Feet in Teenagers. J. Foot Ankle Res. 7, 754. 10.1186/s13047-014-0057-2 25558289 PMC4282742

[B78] Powers C. M. (2010). The Influence of Abnormal Hip Mechanics on Knee Injury: A Biomechanical Perspective. J. Orthop. Sports Phys. Ther. 40, 42–51. 10.2519/jospt.2010.3337 20118526

[B79] Sinclair J. Taylor P. J. Vincent H. (2014). Multisegment Foot Kinematics and Plantar Fascia Strain during Treadmill and Overground Running. Foot Ankle Online J. 7. 10.3827/faoj.2014.0704.0004

[B80] Snyder K. R. Earl J. E. O’Connor K. M. Ebersole K. T. (2009). Resistance Training Is Accompanied by Increases in Hip Strength and Changes in Lower Extremity Biomechanics during Running. Clin. Biomech. 24, 26–34. 10.1016/j.clinbiomech.2008.09.009 19013697

[B81] Taddei U. T. Matias A. B. Duarte M. Sacco I. C. N. (2020b). Foot Core Training to Prevent Running-Related Injuries: A Survival Analysis of a Single-Blind, Randomized Controlled Trial. Am. J. Sports Med. 48, 3610–3619. 10.1177/0363546520969205 33156692

[B82] Taddei U. T. Matias A. B. Ribeiro F. I. A. Bus S. A. Sacco I. C. N. (2020a). Effects of a Foot Strengthening Program on Foot Muscle Morphology and Running Mechanics: A Proof-Of-Concept, Single-Blind Randomized Controlled Trial. Phys. Ther. Sport 42, 107–115. 10.1016/j.ptsp.2020.01.007 31962191

[B83] Taddei U. T. Matias A. B. Ribeiro F. I. A. Inoue R. S. Bus S. A. Sacco I. C. N. (2018). Effects of a Therapeutic Foot Exercise Program on Injury Incidence, Foot Functionality and Biomechanics in Long-Distance Runners: Feasibility Study for a Randomized Controlled Trial. Phys. Ther. Sport 34, 216–226. 10.1016/j.ptsp.2018.10.015 30388670

[B84] Takabayashi T. Edama M. Yokoyama E. Kanaya C. Kubo M. (2018). Quantifying Coordination Among the Rearfoot, Midfoot, and Forefoot Segments during Running. Sports Biomech. 17, 18–32. 10.1080/14763141.2016.1271447 28632051

[B85] Tiberio D. (1987). The Effect of Excessive Subtalar Joint Pronation on Patellofemoral Mechanics: A Theoretical Model. J. Orthop. Sports Phys. Ther. 9, 160–165. 10.2519/jospt.1987.9.4.160 18797010

[B86] van der Worp M. P. ten Haaf D. S. M. van Cingel R. de Wijer A. Nijhuis-Van Der Sanden M. W. G. Staal J. B. (2015). Injuries in Runners; a Systematic Review on Risk Factors and Sex Differences. PLoS One 10, e0114937. 10.1371/journal.pone.0114937 25706955 PMC4338213

[B87] Van Gent R. N. Siem D. van Middelkoop M. Van Os a. G. Bierma-Zeinstra S. M. A. Koes B. W. (2007). Incidence and Determinants of Lower Extremity Running Injuries in Long Distance Runners: a Systematic Review * COMMENTARY. Br. J. Sports Med. 41, 469–480. 10.1136/bjsm.2006.033548 17473005 PMC2465455

[B88] Watari R. Suda E. Y. Santos J. P. S. Matias A. B. Taddei U. T. Sacco I. C. N. (2021). Subgroups of Foot-Ankle Movement Patterns Can Influence the Responsiveness to a Foot-Core Exercise Program: A Hierarchical Cluster Analysis. Front. Bioeng. Biotechnol. 9, 1–13. 10.3389/fbioe.2021.645710 PMC8217875 34169063

[B89] Wearing S. C. Smeathers J. E. Urry S. R. Hennig E. M. Hills A. P. (2006). The Pathomechanics of Plantar Fasciitis. Sports Med. 36, 585–611. 10.2165/00007256-200636070-00004 16796396

[B90] Weist R. Eils E. Rosenbaum D. (2004). The Influence of Muscle Fatigue on Electromyogram and Plantar Pressure Patterns as an Explanation for the Incidence of Metatarsal Stress Fractures. Am. J. Sports Med. 32, 1893–1898. 10.1177/0363546504265191 15572318

[B91] Willems T. M. De Clercq D. Delbaere K. Vanderstraeten G. De Cock A. Witvrouw E. (2006). A Prospective Study of Gait Related Risk Factors for Exercise-Related Lower Leg Pain. Gait & Posture 23, 91–98. 10.1016/j.gaitpost.2004.12.004 16311200

[B92] Williams III D. S. McClay I. S. Hamill J. (2001). Arch Structure and Injury Patterns in Runners. Clin. Biomech. 16, 341–347. 10.1016/S0268-0033(01)00005-5 11358622

[B93] Willy R. W. Davis I. S. (2011). The Effect of a Hip-Strengthening Program on Mechanics during Running and during a Single-Leg Squat. J. Orthop. Sports Phys. Ther. 41, 625–632. 10.2519/jospt.2011.3470 21765220

[B94] Wong Y. S. (2007). Influence of the Abductor Hallucis Muscle on the Medial Arch of the Foot: a Kinematic and Anatomical Cadaver Study. Foot Ankle Int. 28, 617–620. 10.3113/FAI.2007.0617 17559771

[B95] Yeap J. S. Singh D. Birch R. (2001). Tibialis Posterior Tendon Dysfunction: A Primary of Secondary Problem? Foot Ankle Int. 22, 51–55. 10.1177/107110070102200108 11206823

[B96] Zadpoor A. A. Nikooyan A. A. (2011). The Relationship between Lower-Extremity Stress Fractures and the Ground Reaction Force: A Systematic Review. Clin. Biomech. 26, 23–28. 10.1016/j.clinbiomech.2010.08.005 20846765

