# Supplementary material for: Effects of Foot-Core Training on Foot-Ankle Kinematics and Running Kinetics in Runners: Secondary Outcomes From a Randomized Controlled Trial
Source: Front Bioeng Biotechnol. 2022 Apr 14;10:890428. doi: 10.3389/fbioe.2022.890428 (PMC9046605; doi:10.3389/fbioe.2022.890428)
Supplement: Supplementary file 1 [file DataSheet1.PDF]

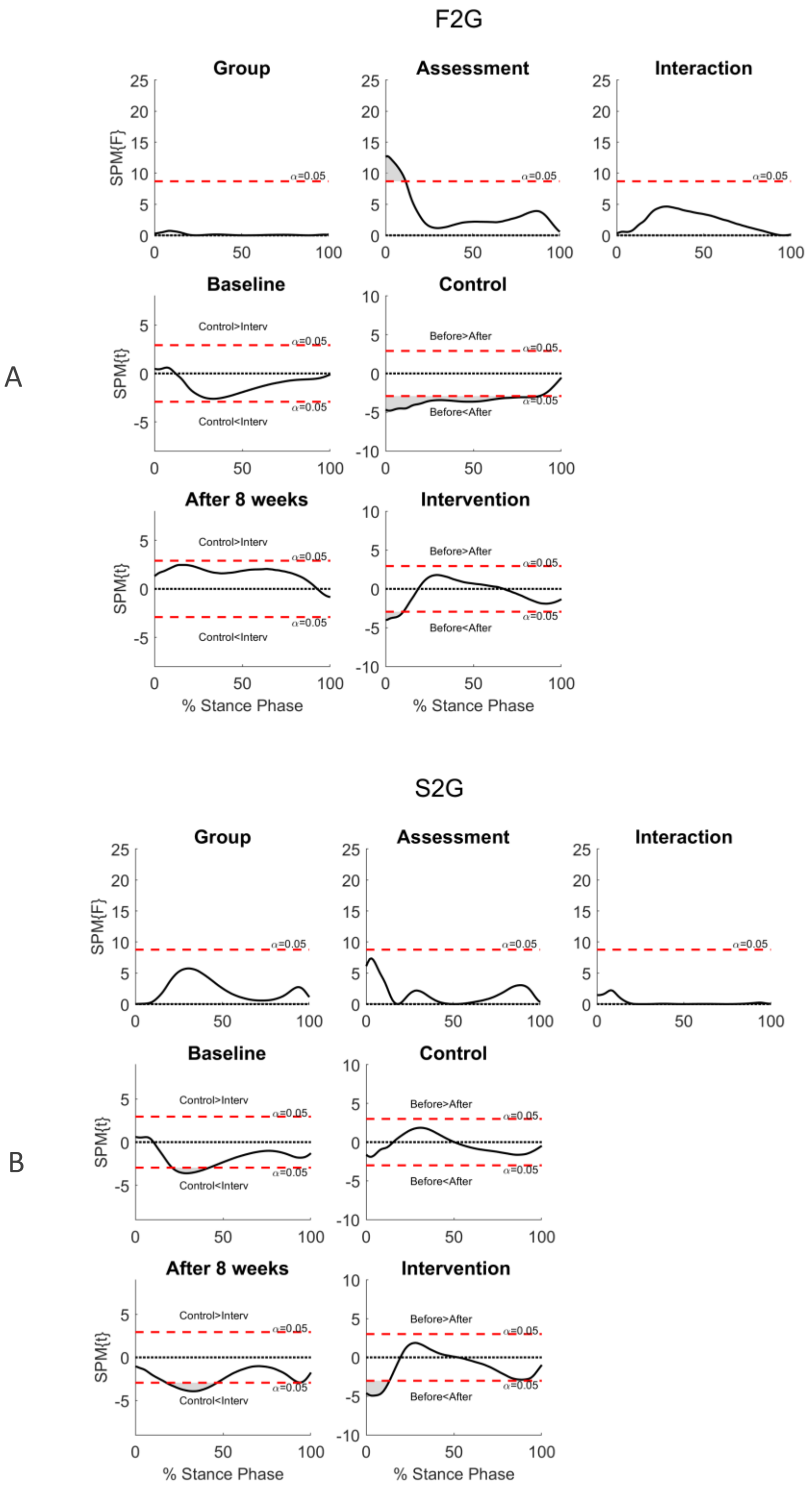

Supplementary Figure 1- Statistical parametric mapping results of F2G (sagittal-plane inclination of first metatarsal bone to the ground **(A)** and S2G (second metatarsal bone to the ground **(B).** Shaded areas indicate significant differences between both waveforms, where the SPM{t} values exceeded the Sidák corrected alpha level threshold.

A

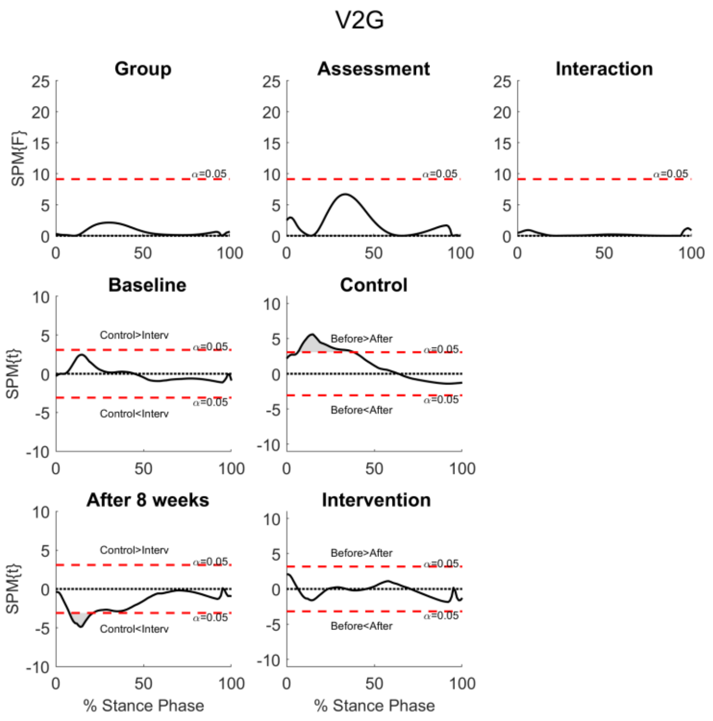

B

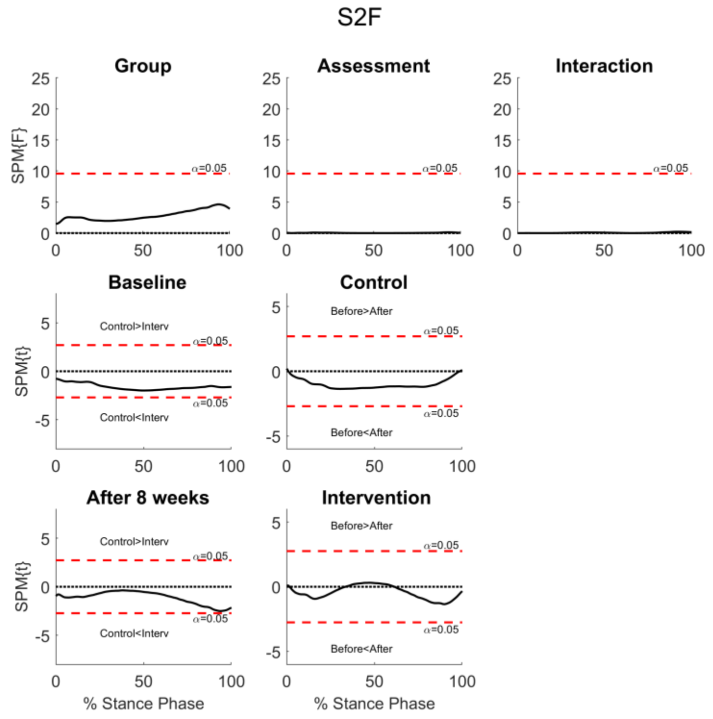

Supplementary Figure 2 - Statistical parametric mapping results of sagittal-plane inclination of V2G (fifth metatarsal bone to the ground) **(A)** and transverse-plane divergence between S2F (first and second metatarsal bones) **(B).** Shaded areas indicate significant differences between both waveforms, where the SPM{t} values exceeded the Sidák corrected alpha level threshold.

A

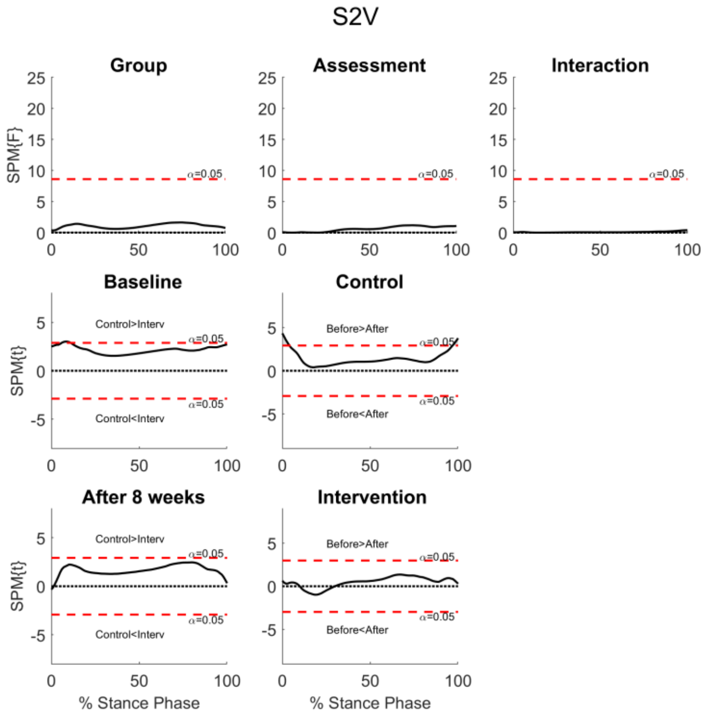

B

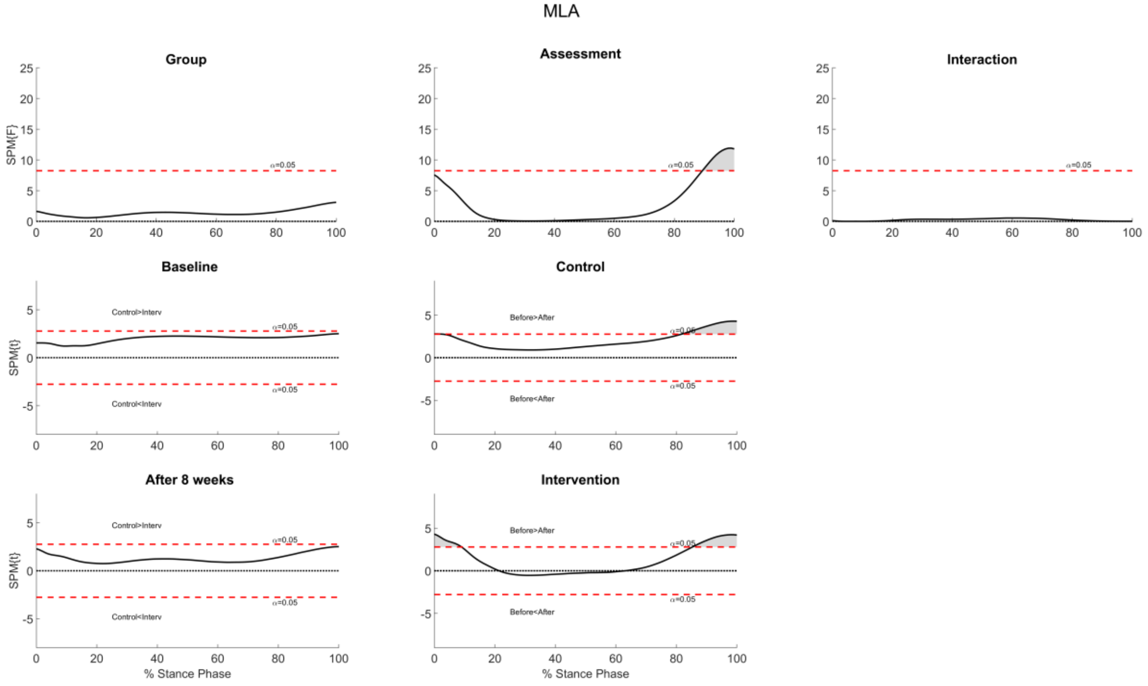

Supplementary Figure 3 - Statistical parametric mapping results of transverse-plane divergence between S2V (second and fifth metatarsal bones) **(A)**, and MLA (medial longitudinal arch) **(B).** Results were normalized across stance phase (0–100%). Shaded areas indicate significant differences between both waveforms, where the SPM{t} values exceeded the Sidák corrected alpha level threshold.

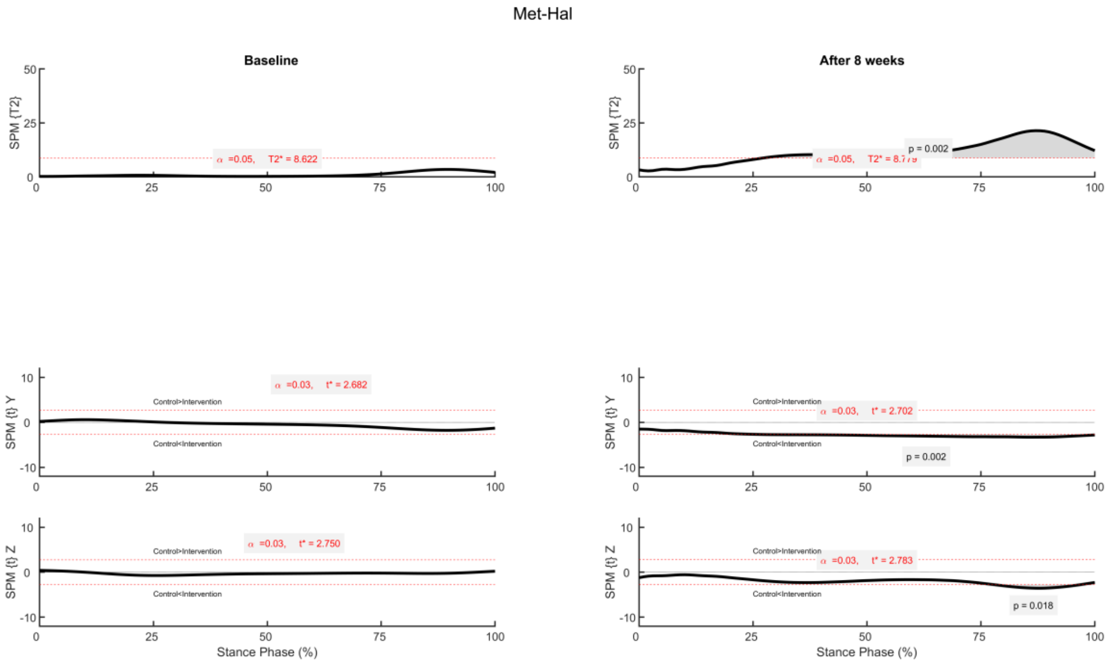

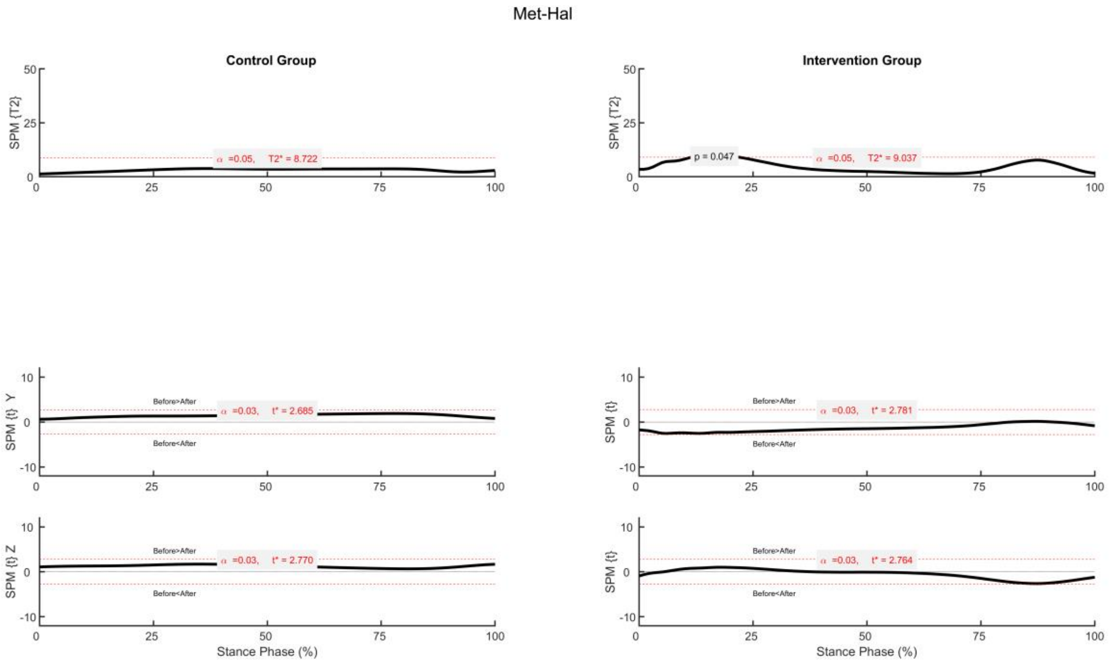

Supplementary Figure 4 - Statistical parametric mapping (3D vector field SPM analysis) followed by the paired or independent t-test of transverse and sagittal plane, as a post-hoc tests, results of transverse-plane divergence and sagittal-plane inclination between first metatarsus and hallux angle (Met-Hal). joint angles time normalized across stance phase (0–100%). Shaded areas indicate significant differences between both waveforms, where the SPM{t} values exceeded the Sidák corrected alpha level threshold.

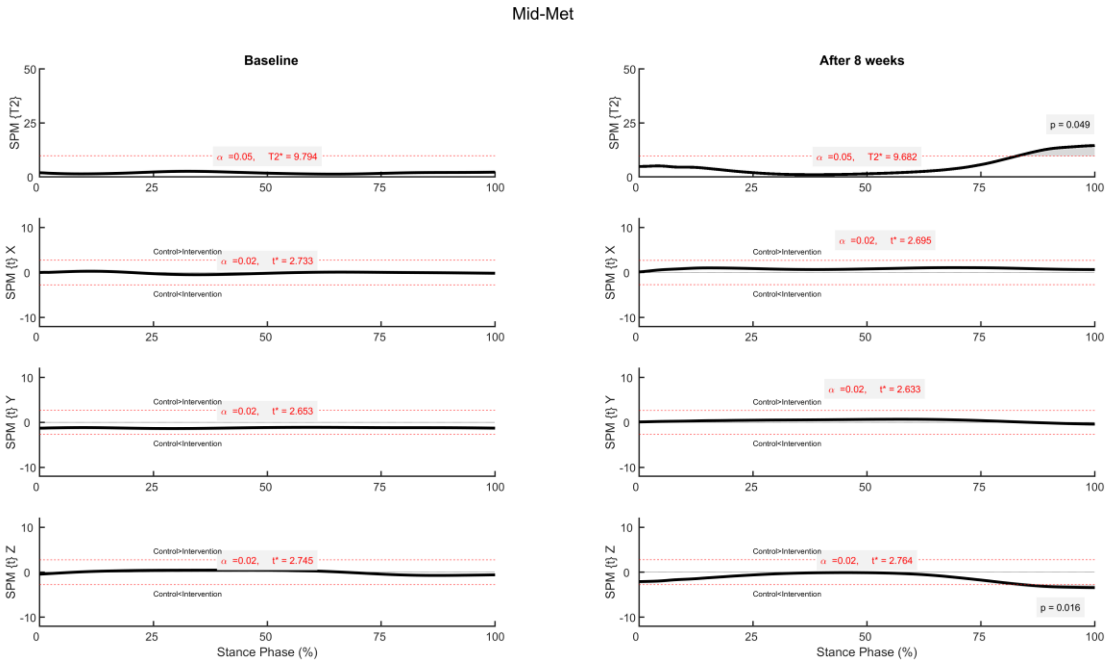

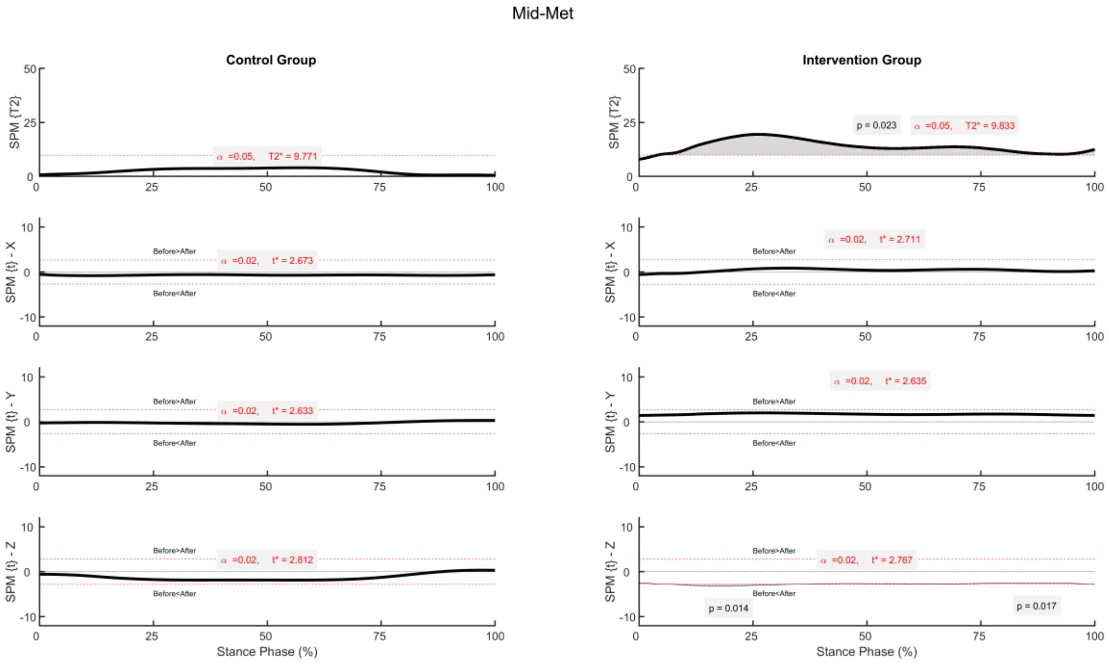

Supplementary Figure 5 - Statistical parametric mapping (3D vector field SPM analysis) followed by the paired or independent t-test of frontal, transverse and sagittal plane, as a post-hoc tests, results of metatarsus with respect to the midfoot (Mid-Met) joint angles time normalized across stance phase (0–100%). Shaded areas indicate significant differences between both waveforms, where the SPM{t} values exceeded the Sidák corrected alpha level threshold.

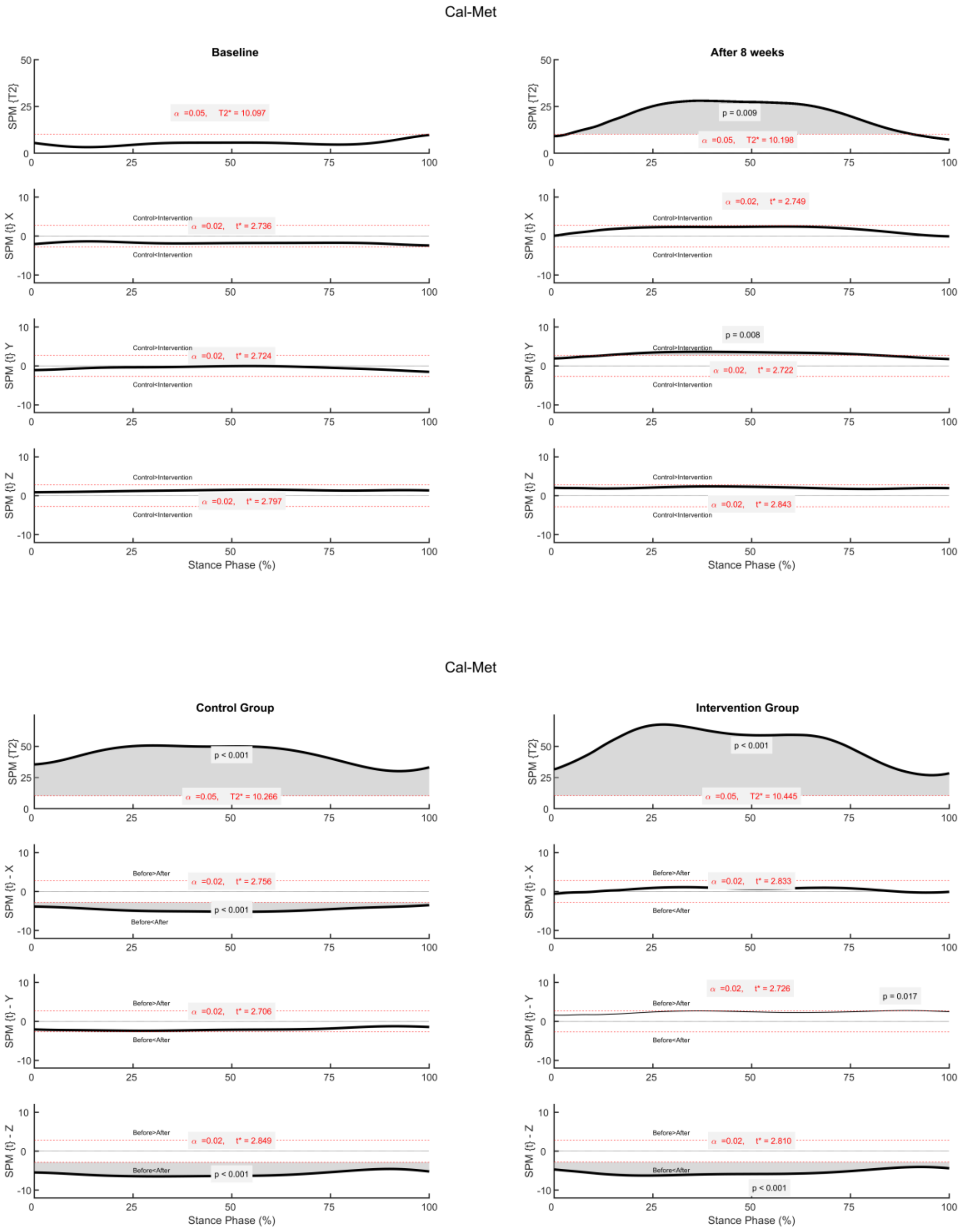

Supplementary Figure 6 - Statistical parametric mapping (3D vector field SPM analysis) followed by the paired or independent t-test of frontal, transverse and sagittal plane, as a post-hoc tests, results of metatarsus with respect to the calcaneus (Cal-Met) joint angles time normalized across stance phase (0–100%). Shaded areas indicate significant differences between both waveforms, where the SPM{t} values exceeded the Sidák corrected alpha level threshold.

Cal-Mid

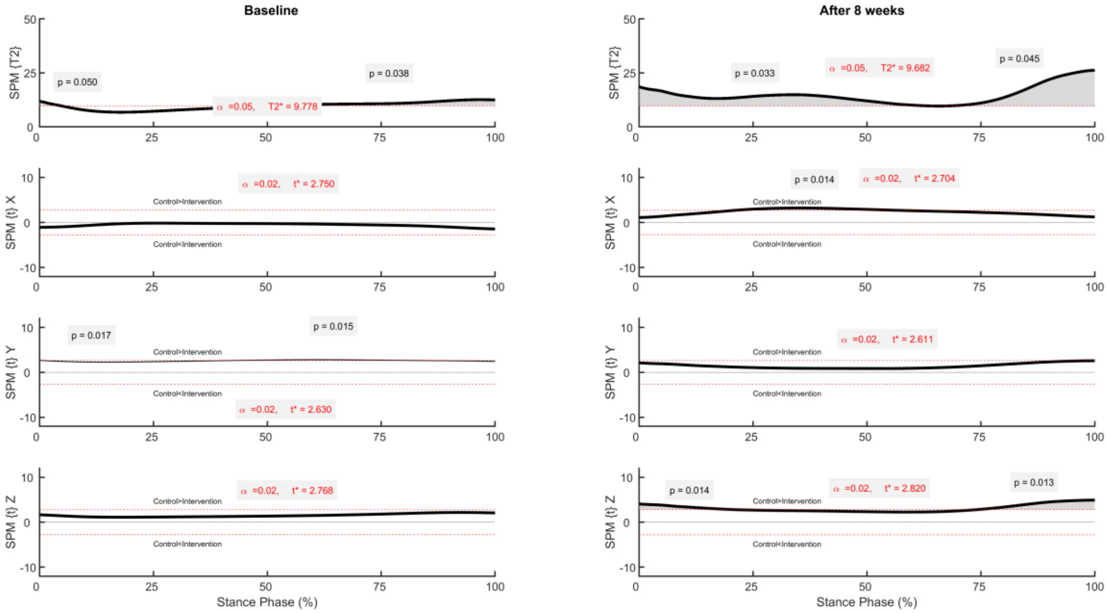

Cal-Mid

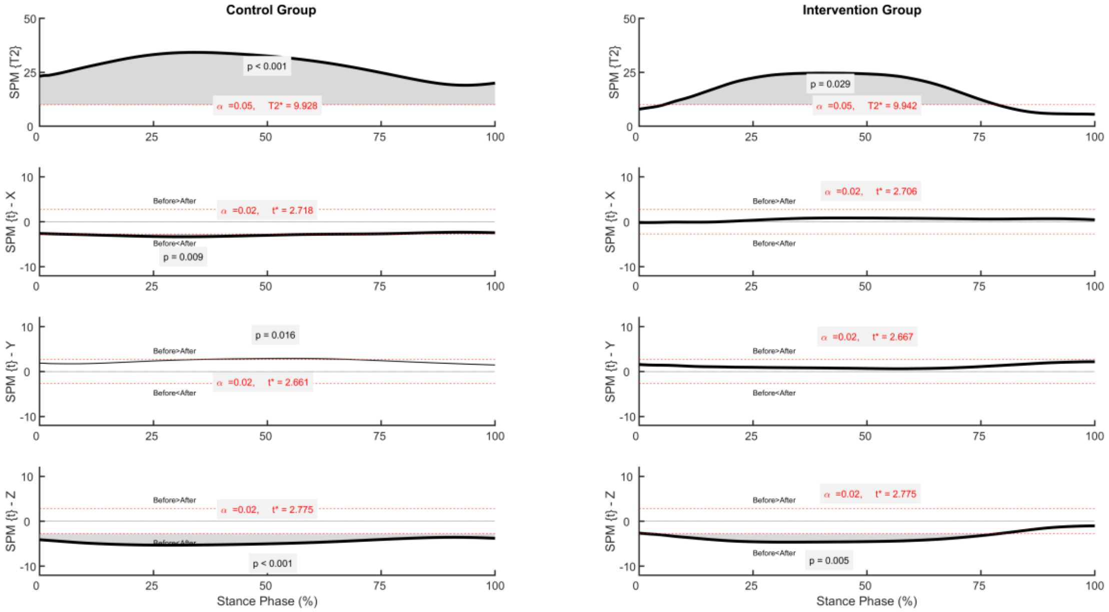

Supplementary Figure 7 - Statistical parametric mapping (3D vector field SPM analysis) followed by the paired or independent t-test of frontal, transverse and sagittal plane, as a post-hoc tests, results of midfoot with respect to the calcaneus (Cal-Mid) joint angles time normalized across stance phase (0–100%). Shaded areas indicate significant differences between both waveforms, where the SPM{t} values exceeded the Sidák corrected alpha level threshold.

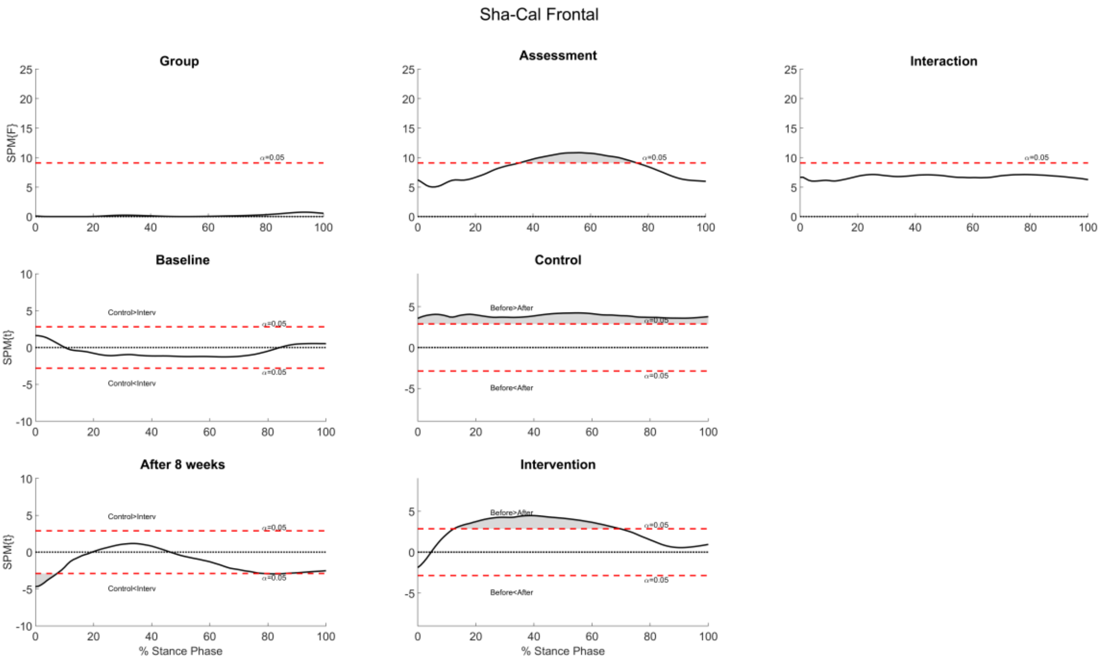

Supplementary Figure 8 - Statistical parametric mapping results of frontal plane between calcaneus with respect to the shank (Sha-Cal) joint angles time normalized across stance phase (0–100%). Shaded areas indicate significant differences between both waveforms, where the SPM{t} values exceeded the Sidák corrected alpha level threshold.

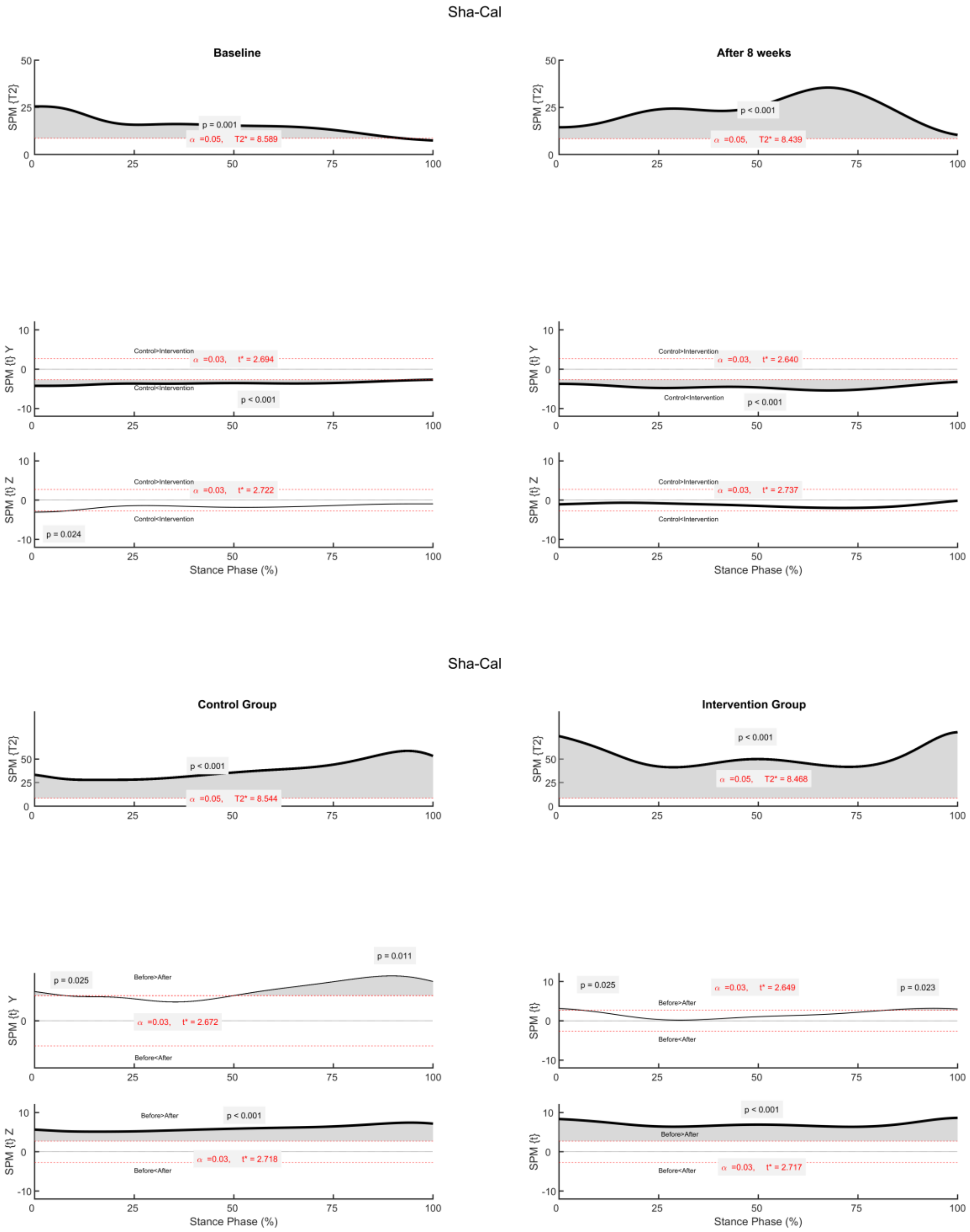

Supplementary Figure 9- Statistical parametric mapping (3D vector field SPM analysis) followed by the paired or independent t-test of transverse and sagittal plane, as a post-hoc tests, results of calcaneus with respect to the shank (Sha-Cal) joint angles time normalized across stance phase (0–100%). Shaded areas indicate significant differences between both waveforms, where the SPM{t} values exceeded the Sidák corrected alpha level threshold.
